# Characterisation of a cold‐adapted, thermostable glucokinase from psychrophilic *Pseudoalteromonas* sp. AS‐131 reveals how the enzyme achieves high thermal stability without loss of cold adaptation

**DOI:** 10.1111/febs.70367

**Published:** 2025-12-15

**Authors:** Akane Yato, Yuki Kato, Fuyuko Hayashi, Rio Asaka, Atsuko Ogawa, Tokuro Oda, Sayaka Tsuji, Masashi Unno, Nobuaki Soh, Keiichi Watanabe, Masaki Horitani

**Affiliations:** ^1^ The United Graduate School of Agricultural Sciences Kagoshima University Japan; ^2^ Department of Applied Biochemistry and Food Science, Faculty of Agriculture Saga University Japan; ^3^ Department of Chemistry and Applied Chemistry, Faculty of Science and Engineering Saga University Japan

**Keywords:** cold‐adapted enzyme, disulfide bond, extremophile, protein engineering, thermal stability

## Abstract

Microorganisms living in cold environments such as the Antarctic and deep sea usually possess cold‐adapted enzymes, which are known to have high catalytic efficiency and low stability owing to their flexible structures. Research on cold‐adapted enzymes has not progressed much due to the challenge of these enzymes being less stable. However, several cold‐adapted enzymes with high thermal stability have recently been reported. In this study, we investigated the biochemical properties of glucokinases from the psychrophilic *Pseudoalteromonas* sp. AS‐131 (PsGK) isolated from the Antarctic Ocean and mesophilic *Escherichia coli* (EcGK). We demonstrated that PsGK is a cold‐adapted enzyme with high thermal stability. A comparison of the crystal structures and spectroscopic studies revealed that PsGK has an additional disulfide bond connecting the N and C termini. To test whether this bond is important for stability, we prepared a PsGK variant by removing the bond and observed the significant reduction in thermal stability. In addition, the introduction of the artificial disulfide bonds in homologous positions in EcGK increased the thermal stability without the reduction in maximum activity. These results confirmed that the introduction of a disulfide bond at the proper position, such as the connection of the N and C termini, significantly improved stability without changing the nature of enzymes. Our findings propose a new strategy that will contribute to the industrial application of enzymes.

## Graphical abstract

We investigated glucokinase from the psychrophilic *Pseudoalteromonas* sp. AS‐131 (PsGK), which was isolated from the Antarctic Ocean. Biochemical studies revealed that PsGK is a cold‐adapted enzyme with high thermal stability, in contrast to general cold‐adapted enzymes, which have low thermal stability. Comparing PsGK with the homologous glucokinase and conducting mutagenesis studies showed that the disulfide bond connecting the N and C termini improves thermal stability without compromising cold adaptation.

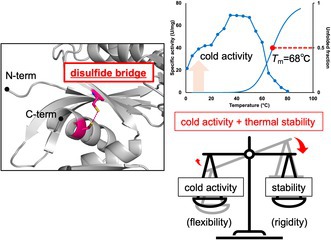

**Abbreviations**
CD: circular dichroism
CpGK: glucokinase from *Colwellia psychrerythraea*
DS‐L: EcGK C20S C65S L313C
DS‐S: EcGK C20S C65S S309C
DTNB: 5′5′‐dithiobis2‐nitrobenzonate
DTT: dithiothreitol
*E. coli*
: *Escherichia coli*
*E*_a_
: activation energy
EcGK: glucokinase from *Escherichia coli*
EcGK DS‐H: EcGK C20S C65S H312C variant
*fpa*
: alkaline protease gene
G6PD: d‐glucose‐6‐phosphate dehydrogenase
*glk*
: glucokinase gene
GRAVY: the grand average hydrophobicity
IPTG: isopropyl‐1‐thio‐β‐*d*‐galactopyranoside
*k*_cat_
: substrate turnover
*k*_cat_/*K*_m_
: catalytic efficiency
*k*_d_
: thermal deactivation constant
*K*_m_
: affinity
MD: molecular dynamics
NADP^+^
: nicotinamide adenine dinucleotide phosphate
PCA: principal component analysis
PhGK: glucokinase from *Pseudoalteromonas haloplanktis*
PsGK: glucokinase from *Pseudoalteromonas* sp. AS‐131
RSA: relative solvent‐accessible surface area
S/V ratio: surface area to volume ratio
SASA: solvent‐accessible surface area
SDS/PAGE: sodium dodecyl sulfate/polyacrylamide gel electrophoresis
SG: cysteine sulfur
*T*_50_
: half‐inactivation time
TcGK: glucokinase from *Trypanosoma cruzi*
*T*_m_
: unfolding mid‐point temperature
TNB: 5‐mercapto‐2‐nitrobenzoic acid
*T*_opt_
: optimum temperature
WT: wild‐type
Δ*H* ^#^
: enthalpy
Δ*S* ^#^
: entropy

## Introduction

Various microorganisms survive in extreme environments, for example at high or low temperatures, high pressure, high salt concentrations and strong acidic or alkaline conditions [1, 2, 3]. Such organisms are the so‐called extremophiles and have evolved by developing adaptive strategies suited to their survival. Enzymes of extremophiles are considered to have many potential applications in the fields of food processing, chemicals, medicine, environmental protection, biotechnology and basic research in molecular biology [4]. The temperature in the Arctic and Antarctic oceans and deep sea is approximately 4 °C [5]. Among extremophiles, Antarctic marine microorganisms are permanently exposed to low temperatures and retain enzymes that have relatively high activity at low temperatures to survive [6, 7]. These enzymes are known to be cold‐adapted and have been isolated from a variety of organisms that live in cold environments, and their biochemical properties have been elucidated [4, 8, 9]. Cold‐adapted enzymes typically exhibit high catalytic efficiency to overcome the reduction in chemical reaction rates caused by low temperatures [10, 11, 12]. This can be achieved by enhancing specific conformational flexibility in regions such as the active site or surface of the protein [9, 13]. However, a side effect of increased flexibility is a decrease in heat resistance; that is, the enzyme is denatured and inactivated at relatively low temperatures compared to homologous mesophilic and thermostable enzymes [9, 14]. Due to their extreme instability, it is difficult to fully realise the industrial use of cold‐adapted enzymes [4]. On the other hand, because thermostable enzymes are highly resistant to proteolysis and chemical denaturation, much research has been conducted on them, and their applications in industrial fields have been progressing [15]. However, in recent years, several psychrophilic enzymes have been reported to have the same or higher stability than their homologous mesophilic counterparts, raising questions about the intrinsic nature of cold‐adapted enzymes [16, 17, 18, 19]. For industrial enzyme applications, both high thermal stability and high activity over a wide range of temperatures are required. The use of enzymes is limited to a small temperature range because most enzymes have low activity at cold temperatures and no activity at high temperatures. Therefore, the development of enzymes with high stability and activity over a wide temperature range is desirable. If we can develop a strategy to artificially modify the functions of enzymes and improve their thermal stability with high catalytic activity, the industrial use of enzymes could be expanded. However, in many cases, these attempts have been unsuccessful. To overcome this challenge, the intrinsic nature of enzymes must be understood. Therefore, further studies on cold‐adapted enzymes that originally possess high activity are required.

The stability of protein conformation is characterised by hydrogen bonds, ion pairs, disulfide bonds and hydrophobic interactions [20, 21, 22]. In particular, disulfide bonds, which are covalent intra‐ and intermolecular interactions, have a strong stabilising effect; thus, naturally occurring disulfide bonds improve protein stability because they reduce the entropy of the unfolded state [23, 24]. Therefore, artificially introducing novel disulfide bonds into proteins is an attractive strategy for improving protein stability. Although it has been reported that the introduction of artificial disulfide bonds can significantly improve the unfolding mid‐point temperature (*T*_m_) [25], in some cases, the introduction of disulfide bonds leads to protein instability, and this attempt has not been fully successful [22, 26].

In addition to disulfide bond formation, various intramolecular interactions are known to contribute to the enhancement of the thermal stability of cold‐adapted enzymes. For instance, the formation of ionic interactions between positively and negatively charged amino acid residues has been reported to stabilise the three‐dimensional structure of proteins [27]. The binding of metal ions has also been shown to increase local structural rigidity and contribute to improved thermal stability [28]. Furthermore, the formation of oligomeric structures through subunit–subunit interactions is an effective strategy for enhancing structural stability, since oligomeric forms are generally more resistant to proteolytic degradation and thermal denaturation than monomers [29]. These multiple stabilising mechanisms may act together to enable cold‐adapted enzymes to maintain a certain degree of thermal stability while retaining high catalytic activity at low temperatures.

Here, we report the biochemical characterisation of glucokinases from the psychrophilic *Pseudoalteromonas* sp. AS‐131 (PsGK), isolated from the Antarctic Ocean. A comparative analysis with the homologous mesophilic glucokinase from *Escherichia coli* K‐12 strain (EcGK) revealed that PsGK is a cold‐adapted enzyme with higher thermal stability than EcGK. To identify the mechanism of high stability with cold adaptation, a comparison of amino acid sequences, crystal structures, circular dichroism (CD) spectroscopy and mutagenesis revealed that a disulfide bond connecting the N and C termini in PsGK contributes to high thermal stability without loss of cold adaptation. In addition, the introduction of a homologous disulfide bond into EcGK increased the *T*_m_, indicating that disulfide bonds at such positions improve the stability of the enzyme. Our findings provide new insights for the design of industrial enzymes.

## Results and discussion

### Selection of PsGK comparisons

**Fig. 1:**
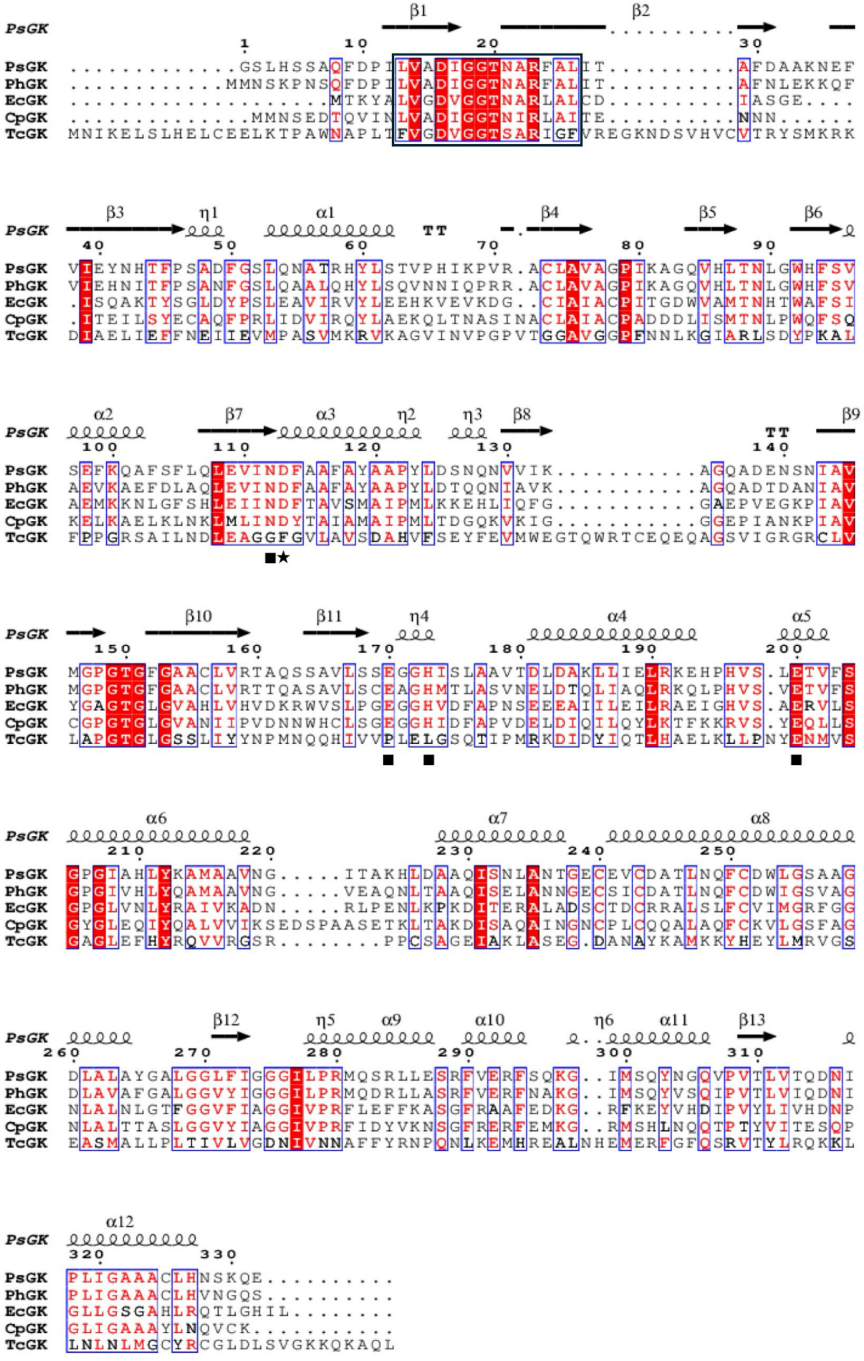
Sequence alignment of glucokinase from *Pseudoalteromonas* sp. AS‐131 (PsGK), glucokinase from *Escherichia coli* (EcGK), glucokinase from *Pseudoalteromonas haloplanktis* (PhGK), glucokinase from *Colwellia psychrerythraea* (CpGK), and glucokinase from *Trypanosoma cruzi* (TcGK). Sequence alignment was performed using clustalw software [85]. The identical residues are shaded in red. The similar amino acid residues are boxed. Strict β‐turns are shown as letters TT. Residues associated with glucose binding (black squares) and catalytic Asp (black star) are indicated. The conserved β‐strand‐loop‐β‐strand motif associated with ATP‐binding (Leu13‐Leu26) is highlighted by black box. The secondary structure elements (α‐helices and β‐strands) of PsGK are depicted above the sequence alignment. This figure was prepared using the espript software [86]. The sequences were obtained from Uniprot with the following accession numbers: PsGK: H7CHS4; EcGK: P0A6V8; PhGK: A0ABU1B9R8; CpGK: Q47XU3; TcGK: Q4E4E1.

Protein family classification showed that PsGK is a member of the group II glucokinase family (PFAM accession number PF02685), similar to EcGK [30]. Figure 1 showed the sequence alignment of PsGK, EcGK, glucokinase from *Pseudoalteromonas haloplanktis* (PhGK), glucokinase from *Colwellia psychrerythraea* (CpGK) and glucokinase from *Trypanosoma cruzi* (TcGK), which belong to the same family shared 38, 76, 40, and 22% sequence identity with PsGK, respectively. Among these, EcGK and TcGK have been extensively characterised with crystal structures and biochemical properties [31, 32, 33] and only TcGK has been reported to exist in equilibrium between monomeric and dimeric states depending on the protein concentration [33]. As mentioned in the introduction, oligomerisation is significantly enhanced thermal stability. Additionally, TcGK has less similarity with PsGK than EcGK. Thus, EcGK was selected as the reference for comparison in this study.

### Purification of PsGK WT and EcGK WT

PsGK and EcGK dialysed after precipitation with ammonium sulfate at 20–50% and 30–60% saturation, respectively, were applied to anion‐exchange chromatography. The activities of PsGK and EcGK were detected from the chromatographic peaks in the range of 150–250 mm and 50–150 mm KCl concentrations on the linear gradient of KCl, respectively (Fig. S1). Sodium dodecyl sulfate/polyacrylamide gel electrophoresis (SDS/PAGE) of the purified enzymes exhibited only one major band with a corresponding molecular mass of approximately 35 kDa (Fig. S2). Furthermore, gel filtration chromatography estimated the molecular weights of PsGK and EcGK to be 79 and 59 kDa, respectively, indicating that both enzymes were homodimers in solution (Fig. S3). These results are consistent with those previously reported for ATP‐dependent glucokinases derived from bacteria and archaea [32, 34, 35].

### Temperature‐dependence on specific activity and thermal stability

**Fig. 2:**
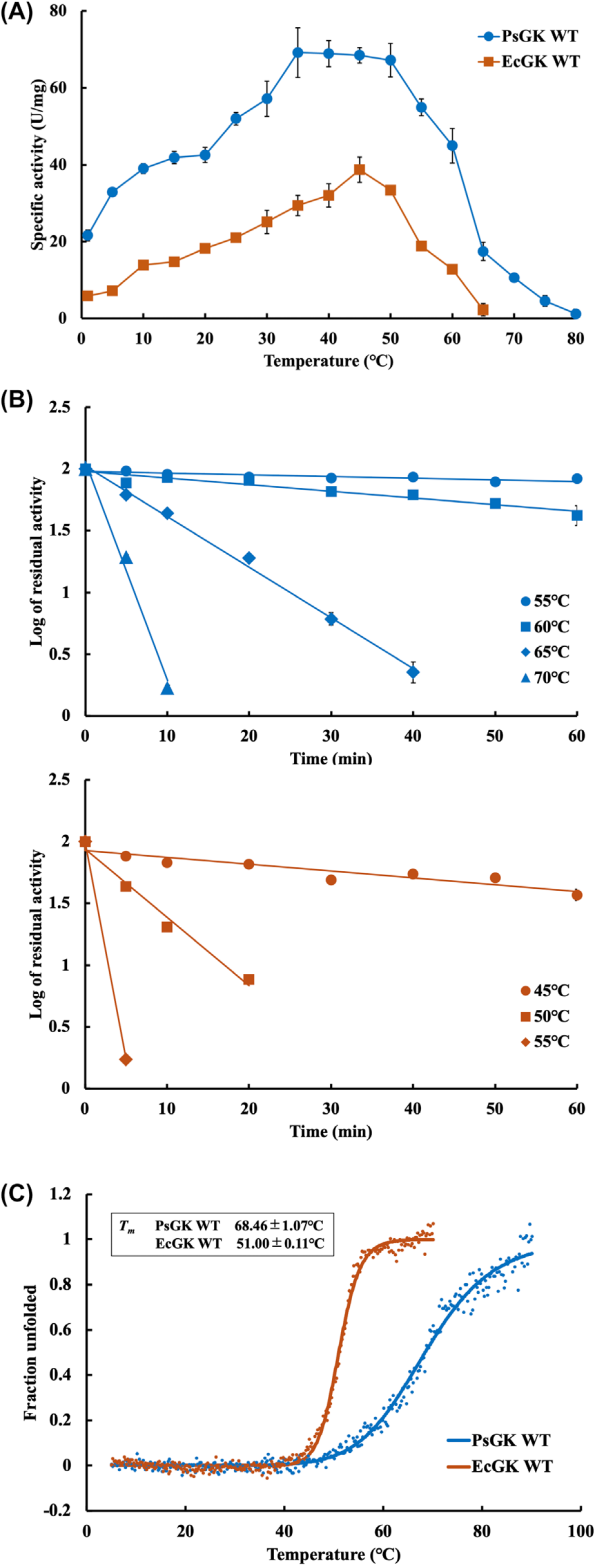
Characterisation of glucokinase from *Pseudoalteromonas* sp. AS‐131 (PsGK) wild‐type (WT) and glucokinase from *Escherichia coli* (EcGK) WT. (A) Plot of specific activity as a function of temperature. The specific activities of PsGK and EcGK were measured at various temperatures. The values represent mean values ± standard deviation of three independent experiments (*n* = 3). (B) Log of residual activities as a function of time of PsGK WT (upper panel) and EcGK WT (lower panel). The residual activities were determined at optimum conditions after pre‐incubation at various temperatures until 60 min. The values represent mean values ± standard deviation of three independent experiments (*n* = 3). (C) Thermal stability examined by thermal melting curves. The change in ellipticity at 222 nm of PsGK WT (blue) and EcGK WT (orange) was monitored as a function of temperature. The measurement temperature was 5–90 °C. The number of accumulations was one.

Figure 2A shows the temperature dependence of the specific activities of PsGK wild‐type (WT) and EcGK WT. The optimum temperature (*T*_opt_) of PsGK and EcGK was 35–50 °C and 45 °C, respectively. The specific activity of PsGK at 1 °C was fourfold higher than that of EcGK, whereas the highest activity of PsGK was twofold higher than that of EcGK. Importantly, the psychrophilic PsGK maintained 31% of its maximum activity at 1 °C, even though the mesophilic EcGK reduced to 15% of its maximum activity at 1 °C (Fig. S4). Psychrophiles typically produce cold‐adapted enzymes that survive in cold environments. Cold‐adapted enzymes are defined as enzymes that optimally catalyse at about 30 °C and maintain some catalytic efficiency at 0 °C [36]. Unlike mesophilic enzymes, they are characterised by the following properties: The optimal catalytic temperature is generally between 20 to 45 °C; the activation energy (*E*_a_) is reduced with increasing substrate turnover (*k*_cat_) or affinity (*K*_m_); thermal stability is low with easily lost activity at high temperatures [4, 9, 37, 38, 39]. Therefore, additional biochemical and biophysical measurements were conducted to determine whether PsGK is a cold‐adapted enzyme.

### Biochemical properties of PsGK WT and EcGK WT

The kinetic parameters of PsGK and EcGK were estimated by steady‐state experiments at 1, 25, and 40 °C (Table 1). The *K*_m_^glucose^ values of PsGK were more favourable than those of EcGK at 1, 25, and 40 °C, while the *K*_m_^ATP^ values of PsGK were less favourable than those of EcGK at 1 and 25 °C. Comparing *K*_m_^glucose^ at 1 °C with those at 25 and 40 °C, PsGK was threefold and fivefold more favourable than at 25 and 40 °C, respectively, whereas EcGK was less favourable than at both 25 and 40 °C. Likewise, comparing *K*_m_^ATP^ at 1 °C with those at 25 and 40 °C, PsGK was approximately twofold more favourable at both 25 and 40°C, and EcGK also exhibited a similar favourabilty. Therefore, it was revealed that PsGK has a higher affinity for glucose than EcGK and its affinity was increased at 1 °C. The specificity constant *k*_cat_/*K*_m_ generally indicates better catalytic efficiency than *k*_cat_ alone [40]. The *k*_cat_/*K*_m_ value of EcGK at 1, 25, and 40 °C decreased as low temperature, exhibiting typical behaviour of a mesophilic enzyme. On the other hand, *k*_cat_/*K*_m_ values of PsGK at 1 °C were significantly greater than those of EcGK. Therefore, PsGK maintained *k*_cat_/*K*_m_ value at low temperatures, consistent with the general characteristics of cold‐adapted enzymes [8].

**Table 1:** Kinetic constants of glucokinase from *Pseudoalteromonas* sp. AS‐131 (PsGK) and glucokinase from *Escherichia coli* (EcGK).

Kinetic constants	PsGK			EcGK		
	1 °C	25 °C	40 °C	1 °C	25 °C	40 °C
*K*_m_^glucose^ (mm)	0.12 ± 0.01	0.36 ± 0.02	0.60 ± 0.11	1.24 ± 0.08	0.73 ± 0.06	0.81 ± 0.15
*K*_m_^ATP^ (mm)	0.83 ± 0.14	1.55 ± 0.17	1.54 ± 0.20	0.78 ± 0.03	1.11 ± 0.08	2.25 ± 0.28
*k*_cat_ (s^−1^)	11.25 ± 0.28	33.49 ± 0.77	40.01 ± 5.23	16.75 ± 0.84	51.36 ± 2.00	70.42 ± 6.88
*k*_cat_/*K*_m_ (mm ^−1^·s^−1^)	92.15 ± 5.17	93.99 ± 3.98	67.93 ± 4.98	13.57 ± 0.21	70.41 ± 3.07	88.18 ± 8.00

The activation energies (*E*_a_) of the reactions catalysed by PsGK and EcGK were determined from the Arrhenius plots shown in Fig. S5. The thermodynamic activation parameters at 25 °C were calculated using the formula described in the Methods section. Cold‐adapted enzymes usually have lower *E*_a_, enthalpy (Δ*H* ^#^), and entropy (Δ*S* ^#^) in catalytic reactions than the corresponding homologous mesophilic enzymes because of their more flexible structure [40, 41]. PsGK exhibited lower *E*_a_, Δ*H* ^#^, and Δ*S* ^#^ than mesophilic EcGK (Table 2).

**Table 2:** Thermodynamic activation constants of glucokinase from *Pseudoalteromonas* sp. AS‐131 (PsGK) and glucokinase from *Escherichia coli* (EcGK).

Thermodynamic activation constants (25°C)	PsGK	EcGK
*E*_a_ (kJ·mol^−1^)	23.56 ± 1.37	31.20 ± 0.83
Δ*G* ^#^ (kJ·mol^−1^)	64.33	63.27
Δ*H* ^#^ (kJ·mol^−1^)	21.08	28.72
Δ*S* ^*#*^ (J·mol^−1^·K^−1^)	−145.08	−115.88

### Thermal stability of PsGK WT and EcGK

We have measured thermal stability using two methods, that is residual activity and CD spectroscopy. Figs 2B and S6 depict the thermal stability determined by the residual activity of PsGK WT and EcGK WT. Residual activities were determined at the optimum conditions after pre‐incubation at various temperatures until 60 min. EcGK retained 37% of its activity after 60 min of heating at 45 °C and it did not show almost no activity above 50 °C, whereas PsGK retained 84 and 43% of its activity after 60 min of heating at 55 and 60 °C, respectively, and it almost completely lost activity above 65 °C (Fig. S6). Table S1 summarised the thermal deactivation constants (*k*_d_) calculated from Fig. 2B. EcGK was inactivated at a rate of 5.6 × 10^−3^ min^−1^ at 45 °C, whereas PsGK exhibited a similar inactivation rate (5.4 × 10^−3^ min^−1^) at higher temperature, 60 °C. These results indicated that PsGK was more thermally stable than EcGK, as it retained activity at higher temperatures.

Next, we monitored the change in the CD spectra with ellipticity at 222 nm. The thermal melting curves as a function of temperature are shown in Fig. 2C. The *T*_m_ values of PsGK WT and EcGK WT were 68.46 ± 1.07 and 51.00 ± 0.11 °C, respectively. Remarkably, PsGK has higher thermal stability than mesophilic EcGK. One of the main features of cold‐adapted enzymes is that thermal inactivation precedes structural unfolding; in other words, enzymatic activity is significantly reduced or lost while the tertiary structure of the enzyme remains largely intact [42, 43]. A useful parameter to describe this unique behaviour is *T*_gap_; difference between *T*_m_ and *T*_opt_, which is typically large in cold‐adapted enzymes [44]. *T*_gap_ of PsGK was 26 °C, which is close to the reported average *T*_gap_ of cold‐adapted enzymes (19.05 ± 2.71 °C), supporting that PsGK can be classified as a cold‐adapted enzyme (Table 3). Furthermore, following a 10‐min thermal incubation at various temperatures, the half‐inactivation time (*T*_50_) of PsGK was determined to be 67.1 °C (Fig. S7). These results indicated that the enzyme retains its functionality even after a 10‐min exposure to temperatures higher than its optimum, reflecting not only its structural stability but also its high functional thermostability. In general, cold‐adapted enzymes maintain a flexible structure, particularly around the active site, in order to achieve high catalytic efficiency at low temperatures. As a result, their thermal stability is often markedly reduced. Many studies have reported that such enzymes exhibit *T*_50_ values significantly lower than their *T*_m_ (*T*_50_ ≪ *T*_m_), indicating that catalytic activity is lost prior to structural denaturation [44, 45]. In contrast, PsGK displayed nearly identical *T*_50_ and *T*_m_ values (*T*_50_ ≈ *T*_m_), suggesting that both structural and functional stability are retained even at elevated temperatures. Such a property is extremely rare among cold‐adapted enzymes and has been scarcely reported in natural systems. Therefore, PsGK represents a highly unusual case, combining two typically incompatible features: catalytic activity at low temperatures and structural and functional stability at high temperatures. Further analyses and experiments were conducted to elucidate the mechanisms underlying the cold adaptation and high thermal stability of PsGK.

**Table 3:** Summary of optimum temperature (*T*_opt_) and unfolding mid‐point temperature (*T*_m_), and their difference (*T*_gap_).

Enzyme^a^	*T*_opt_ (°C)	*T*_gap_ (°C)	*T*_m_ (°C)
PsGK WT	35–50	26	68.46 ± 1.07
EcGK WT	45	6	51.00 ± 0.11
PsGK C325S	40–45	20	62.55 ± 0.32
EcGK DS‐H	n.d.	n.d.	43.09 ± 0.09
EcGK DS‐L	n.d.	n.d.	44.60 ± 0.12
EcGK DS‐S	45	4	49.16 ± 0.09

^a^ PsGK, glucokinase form *Pseudoalteromonas* sp. AS‐131; EcGK, glucokinase from *Escherichia coli*; WT, wild‐type; DS‐H: EcGK C20S C65S H312C; DS‐L: EcGK C20S C65S L313C; DS‐S: EcGK C20S C65S S309C.

### Comparison of amino acid sequences

The amino acid composition of PsGK was compared to that of EcGK (Table 4). Cold‐adapted enzymes exhibit several unique characteristics in their primary structures. Indeed, several researchers have reported that cold‐adapted enzymes maintain their high catalytic activity at low temperatures, mainly because of their more flexible structures achieved by reducing interactions, such as salt bridges, disulfide bonds, hydrogen bonds and hydrophobic interactions [9, 41, 46, 47]. The number of salt bridges estimated from the crystal structures of PsGK and EcGK was 17 and 56, respectively, which revealed that psychrophilic PsGK has fewer salt bridges than the homologous mesophilic EcGK, consistent with general cold‐adapted enzymes [41]. Furthermore, the number of hydrophobic interactions in the crystal structure of psychrophilic PsGK was fewer than in mesophilic EcGK, which is consistent with a general characteristic of cold‐adapted enzymes [48, 49]. However, the grand average hydrophobicity (GRAVY), a hydrophobicity index, of PsGK was comparable to that of EcGK [50, 51]. These findings suggest that the reduced number of salt bridges and hydrophobic interactions in PsGK may contribute to its flexible structure, which in turn enables PsGK to maintain high activity at low temperatures. It has been reported that there is a correlation between the aliphatic index calculated from molar ratios and the relative volumes of the Ala, Val, Ile and Leu residues and the thermal stability of the protein, and a greater index is associated with higher thermal stability [52]. The aliphatic index of PsGK was lower than that of EcGK, suggesting lower thermal stability of PsGK. The presence of Arg residues was estimated from the Arg/(Arg + Lys) ratio. The ratios of PsGK and EcGK were 0.45 and 0.48, respectively, which were not significantly different, although the occurrence of arginine residues was low in cold‐adapted enzymes [53]. Thus, we concluded that the higher thermal stability of PsGK was not due to its amino acid sequence.

**Table 4:** Properties of glucokinase from *Pseudoalteromonas* sp. AS‐131 (PsGK) and glucokinase from *Escherichia coli* (EcGK) determined from a comparison of the deduced amino acid sequences.

GK	Amino acid frequencies (%)^a^						pI	Arg/(Arg + Lys)	GRAVY^b^ index	Aliphatic index
	Charged	Acidic	Basic	Polar	Hydrophobic	Aromatic				
PsGK	18.07	8.43	6.02	23.19	42.47	8.13	5.81	0.45	0.167	95.33
EcGK	24.92	11.53	9.66	20.56	41.12	8.10	6.06	0.48	0.154	101.81

^a^ The charged amino acids are Arg, Lys, His, Asp, and Glu; acidic amino acids are Asp and Glu; basic amino acids are Lys and Arg; polar amino acids are Asn, Cys, Glu, Ser, and Thr; hydrophobic amino acids are Ala, Ile, Leu, Phe, Trp, and Val; aromatic amino acids are Phe, Trp, and Tyr.

^b^ The grand average hydrophobicity.

To investigate the surface characteristics of PsGK and EcGK, we performed structural analysis using FreeSASA [54]. The solvent‐accessible surface area (SASA) and relative solvent‐accessible surface area (RSA) were calculated based on PDB structures for each protein, and the degree of surface exposure of hydrophobic, hydrophilic and charged residues was compared. Residues with RSA values greater than 0.25 were defined as surface‐exposed. The analysis revealed that hydrophobic residues in PsGK and EcGK exhibited comparable surface exposure, with RSA values of 26.67 and 26.35%, respectively. Similarly, the RSA values for hydrophilic residues were 73.33% for PsGK and 73.65% for EcGK, indicating no substantial difference between the two enzymes. It is generally reported that psychrophilic enzymes tend to increase the surface exposure of hydrophobic residues as a strategy to enhance structural flexibility and maintain catalytic activity under cold conditions. The surface exposure of hydrophobic residues in such enzymes typically reaches around 30% RSA [53, 55]. However, PsGK showed a slightly lower value, with almost no difference to EcGK, a mesophilic enzyme. In contrast, the surface exposure of charged residues showed a more distinct difference: 28.89% in PsGK versus 43.32% in EcGK. Charged residues contribute to protein solubility and flexibility through hydration interactions; however, excessive surface exposure of these residues may increase dynamic fluctuations and, in some cases, compromise structural stability [55]. This effect is particularly relevant under cold conditions, where molecular motion is inherently reduced [56]. In such environments, overly flexible structures may lose conformational stability and become more prone to denaturation. These results suggest that PsGK, despite being adapted to cold environments, does not rely on the conventional psychrophilic adaptation strategy of increasing flexibility. Instead, it appears to maintain structural stability by limiting the surface exposure of both hydrophobic and charged residues. This analysis indicates that PsGK may have evolved to balance stability and function without adopting the pronounced surface exposure trends typically observed in psychrophilic enzymes.

Compact protein structures contribute to structural stability. The surface area to volume ratio (S/V ratio), calculated from SASA and molecular volume, serves as an indicator of structural compactness, with higher values reflecting less compact structure. The S/V ratio of PsGK and EcGK was determined to be 0.330 Å^−1^ and 0.329 Å^−1^, respectively, indicating no significant difference in compactness between the two enzymes. Cold‐adapted enzymes are generally characterised by loosened hydrophobic cores and increased surface exposure, which are associated with less compact structures. However, PsGK cannot be clearly defined as less compact than EcGK. Instead, the results suggest that PsGK may represent a balanced form of cold adaptation, achieving both high catalytic activity and structural stability. PsGK retains enzymatic activity at low temperatures without becoming excessively flexible, likely through maintaining a stable hydrophobic core and modulating the distribution of charged and polar residues to prevent structural collapse.

### Presence of a unique disulfide bond involved in thermal stability

**Fig. 3:**
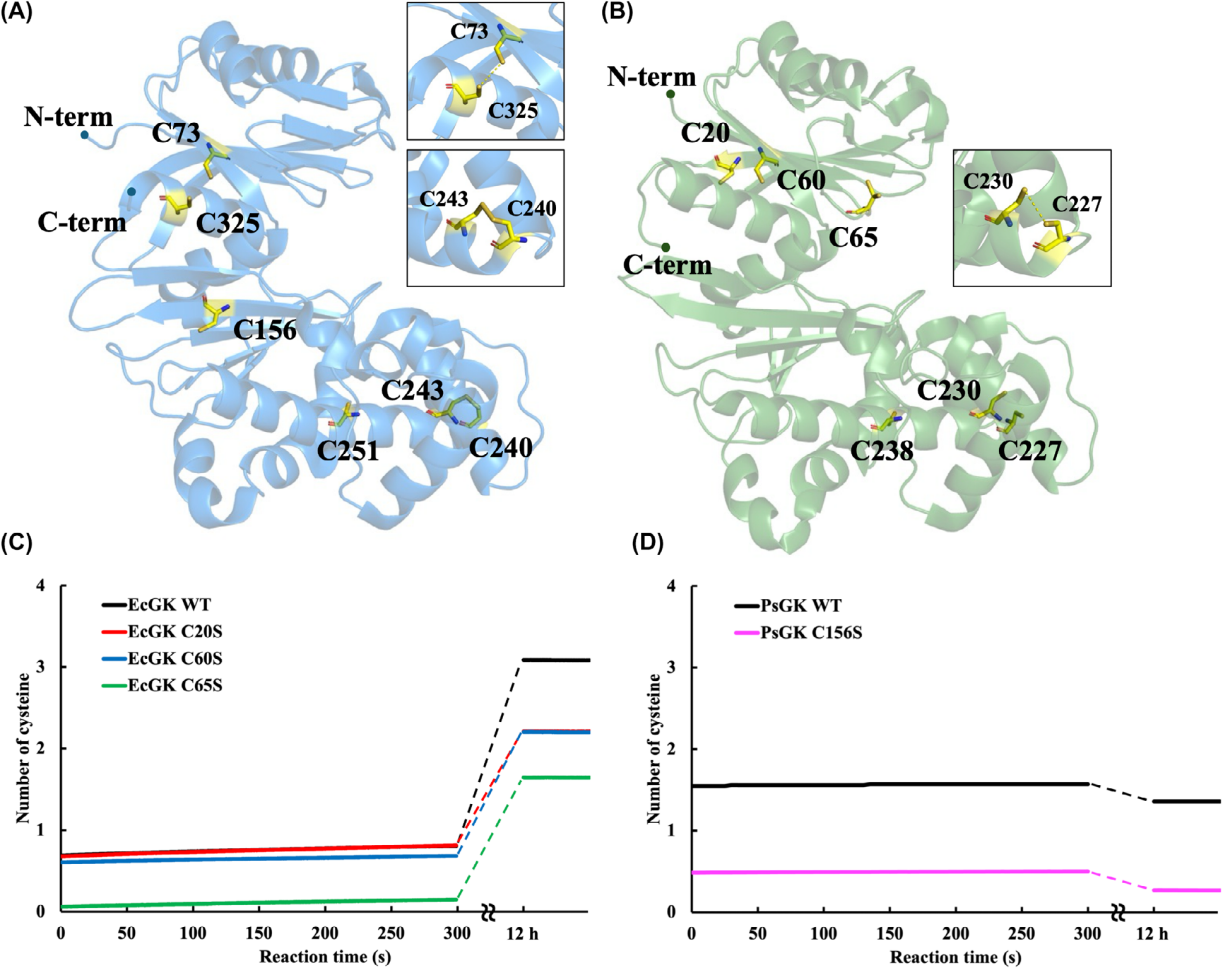
Comparison of crystal structures and time dependence of 5′5’‐dithiobis(2‐nitrobenzonate) (DTNB) reaction for glucokinase from *Pseudoalteromonas* sp. AS‐131 (PsGK) and glucokinase from *Escherichia coli* (EcGK). Crystal structures of (A) PsGK wild‐type (WT) (PDB ID: 3VPZ) and (B) EcGK WT (PDB ID: 1Q18) were shown in blue and green, respectively. The positions of cysteine residues are shown by sticks coloured yellow. Cys73‐Cys325 and Cys240‐Cys243 in PsGK WT and Cys227‐Cys230 in EcGK WT form disulfide bonds. Cys240‐Cys243 in PsGK WT and Cys227‐Cys230 in EcGK are located in homologous positions. The image was prepared using pymol. Comparison of the number of cysteines that reacted with DTNB as a function of time in (C) EcGK and (D) PsGK. Protein solution (PsGK: 6.7 μm, EcGK: 11 μm) were reacted and monitored the absorbance at 412 nm for 5 min. After 12 h later, the absorbance at 412 nm of the reaction mixture was measured. The number of accumulations was one.

We focussed on the role of disulfide bonds and investigated whether PsGK possesses disulfide bonds that contribute to its high thermal stability. Generally, disulfide bonds enhance protein stability by reducing the flexibility and entropy of the unfolded state [57, 58]. By combining the 5′5′‐dithiobis(2‐nitrobenzonate) (DTNB) assay with site‐directed mutagenesis (Cys to Ser), we quantified the number of free cysteine residues in PsGK and EcGK to indirectly determine the number of disulfide bonds formed in a solution. Both PsGK and EcGK contained six cysteine residues in their sequences (Figs 1 and 3A,B). To determine the number of free cysteine residues, PsGK WT and EcGK WT were reacted with DTNB by measuring the change in absorbance at 412 nm as a function of time.

In EcGK WT, one cysteine residue reacted with DTNB immediately after mixing with the enzyme, followed by a gradual reaction of two additional cysteine residues suggesting the possible existence of up to one disulfide bond (Fig. 3C). According to the crystal structure (Fig. 3B), Cys65 and Cys227 were exposed on the surface, indicating that these residues were accessible to DTNB. In the DTNB assay of EcGK C65S, none of cysteine residues reacted immediately with DTNB; instead, two cysteine residues gradually over time (Fig. 3C). This indicated that in EcGK WT, DTNB reacted immediately with Cys65. In both EcGK C20S and EcGK C60S, one cysteine residue (Cys65) reacted with DTNB immediately, and another cysteine residue reacted with DTNB slowly (Fig. 3C). These results indicated that DTNB reacted gradually with Cys20 and Cys60, which are located in the interior of the enzyme. Although Cys227 was exposed on the surface, it did not react with DTNB, which is likely due to form a disulfide bond with the adjacent Cys230, as suggested by the crystal structure (Fig. 3B). Indeed, Cys227‐Cys230 in EcGK is homologous to Cys240‐Cys243, which forms a disulfide bond in the crystal structure of PsGK (Fig. 3A), supporting the likelihood that this disulfide bond also forms in solution. The absence of a clearly defined disulfide bond between Cys227 and Cys230 in the crystal structure of EcGK is likely due to its reduction caused by X‐ray irradiation during data collection. Therefore, it was concluded that EcGK WT possesses one disulfide bond between Cys227 and Cys230.

In PsGK WT, one cysteine residue reacted with DTNB immediately after mixing the enzyme with DTNB, and no additional cysteine residue reacted with DTNB over time, suggesting the possible existence of up to two disulfide bonds (Fig. 3D). Based on the crystal structure (Fig. 3A), Cys73, Cys156 and Cys243 were exposed on the surface of PsGK and were located at a position accessible to DTNB, whereas the rest were located inside the enzyme. We expected either Cys73 or Cys156 to react with DTNB because the crystal structure showed that Cys243 formed a disulfide bond with Cys240 (Fig. 3A inset). We conducted DTNB measurements on the PsGK C156S variant and found that no cysteine residues reacted with DTNB (Fig. 3D). Therefore, Cys156 was the only cysteine residue in PsGK that reacted with DTNB. Interestingly, although Cys73 in PsGK was exposed on the enzyme surface, it did not react with DTNB, whereas Cys60 in EcGK, which is homologous to Cys73 in PsGK, reacted slowly with DTNB. These findings prompted us to consider the possibility that Cys73 forms a disulfide bond with the adjacent Cys325. Generally, the distance between *C*_β_ of cysteine residues forming a disulfide bond is within 5.5 Å [59]. Thus, we expected that Cys73 and Cys325 formed a disulfide bond in solution, as the distance between *C*_β_ of Cys73 and Cys325 in the crystal structure of PsGK was 5.3 Å, although they did not form a disulfide bond in the crystal structure, which could be caused by the reductive cleavage of the bond by X‐ray irradiation. Taken together, we concluded that PsGK possesses not only the Cys227‐Cys230 disulfide bond homologous to that in EcGK but also a unique disulfide bond between Cys73 and Cys325. In general, disulfide bridges affect the stability of proteins [57, 58], and several studies have reported that the introduction of disulfide bonds improves their thermal stability [60, 61, 62, 63]. Interestingly, Cys73 is located on the five parallel strands of the β‐sheet containing the N termini, and Cys325 is located on the α‐helix of C termini in PsGK (Fig. S8a). In addition, these β‐sheets of PsGK have a larger number of hydrogen bonds than those of EcGK (Fig. S8b). We propose that this disulfide bond plays an important role in the tight anchoring of the N termini to the C termini. Thus, we hypothesised that the disulfide bond of Cys73‐Cys325 connected to the N and C termini contributed to the higher thermal stability of PsGK. To test our hypothesis, we prepared a PsGK C325S variant to remove the disulfide bond and measured its thermal stability using CD spectroscopy.

### Contribution of the disulfide bond to high thermal stability of PsGK

**Fig. 4:**
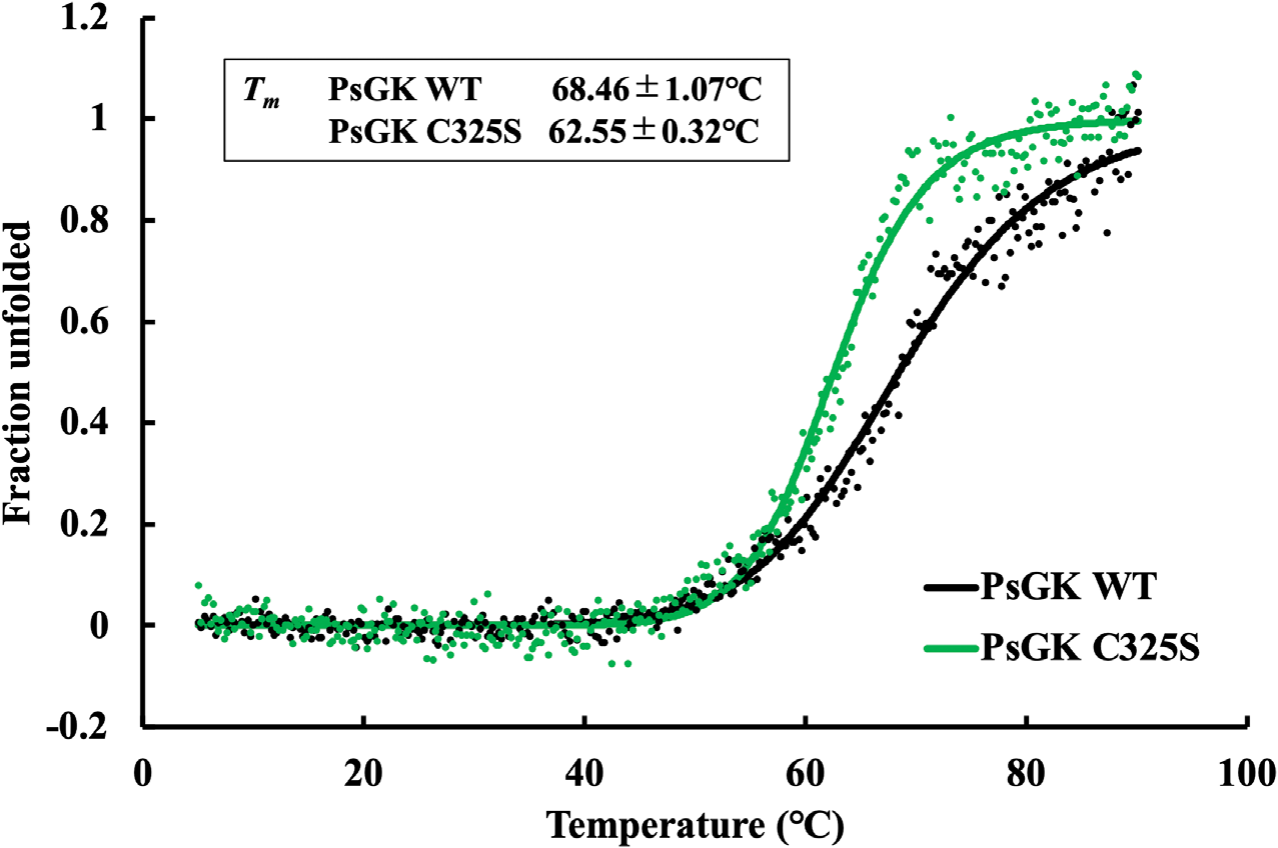
Thermal melting curves of glucokinase from *Pseudoalteromonas* sp. AS‐131 (PsGK) wild‐type (WT) and PsGK C325S. The change in ellipticity at 222 nm of PsGK WT (black) and C325S (green) was monitored as a function of temperature. The measurement temperature was 5–90 °C. The number of accumulations was one. PsGK WT was replotted from the data shown in Fig. 2C for comparison.

Differences in the CD spectra and *T*_m_ values between PsGK WT and PsGK C325S were observed (Fig.4). The *T*_m_ values of PsGK WT and PsGK C325S were 68.46 ± 1.07 and 62.55 ± 0.32 °C, respectively, and the *T*_m_ of PsGK C325S drastically decreased by 6.0 °C, indicating that the thermal stability of PsGK C325S was reduced by the removal of the disulfide bond. These results experimentally demonstrated that such a disulfide bond contributes to the high thermal stability of PsGK. To investigate the effect of disulfide bond formation on enzymatic activity, we compared PsGK WT and the C325S variant (Fig. S9 and Tables 1 and S2). The optimal temperature of C325S was in the range of 40–45 °C, comparable to that of WT. Furthermore, the specific activity of C325S at 1 °C was also comparable to that of WT, indicating that cold adaptation was retained even in the C325S variant. However, the specific activity at 1 °C relative to the maximum activity was approximately 15% for C325S, which was lower than that of WT. In the temperature range of 30–65 °C, C325S exhibited approximately 1.5‐fold higher specific activity than WT. Additionally, the catalytic efficiency (*k*_cat_/*K*_m_) of C325S exceeded that of WT at all tested temperatures (1, 25, and 40 °C). These results suggested that the disulfide bond contributes to the structural stabilisation of PsGK, particularly by maintaining the active conformation under low‐temperature conditions. On the other hand, the removal of the disulfide bond in C325S likely increased structural flexibility, facilitating conformational dynamics in the catalytic process, which in turn led to increased *k*_cat_/*K*_m_ values and specific activities. In other words, the disulfide bond appears to suppress catalytic efficiency to some extent as a trade‐off for ensuring structural stability. As previously reported, in PsGK, the disulfide bond functions as a structural element that regulates the trade‐off between structural stability and catalytic flexibility. In addition, as described in the previous topic, PsGK exhibits remarkably high thermal stability with a *T*_m_ of 68.46 ± 1.07 °C, and furthermore, it was found that *T*_50_ ≈ *T*_m_, a result that contrasted with the typical characteristics of cold‐adapted enzymes. These findings suggest that enzyme activity is not immediately lost even when partial structural denaturation occurs, indicating that the local structure around the active site is relatively robust against thermal stress. Therefore, the disulfide bond is likely to serve a role in selectively stabilising the region surrounding the active site, rather than the overall protein structure, thereby separating structural denaturation from activity loss. Such a function of disulfide bonds aligns well with existing studies on cold‐adapted enzymes. This role of disulfide bonds is consistent with previous findings on cold‐adapted enzymes. D'Amico *et al*. [64] reported that the introduction of a disulfide bond into a cold‐adapted α‐amylase enhanced thermal stability but reduced catalytic activity. The present findings on PsGK support the notion that disulfide bonds act as structural elements that fine‐tune the balance between stability and flexibility. Furthermore, Feller *et al*. [8] demonstrated that high catalytic efficiency in cold‐adapted enzymes arises from increased structural flexibility, but this flexibility compromises overall stability [39, 65]. These findings strongly suggest that disulfide bonds may function as an adaptive evolutionary mechanism to mitigate such trade‐offs.

In addition, we investigated the conservation of the cysteine residues (Cys73 and Cys325) that form the disulfide bond contributing to thermal stability. Sequence alignment among glucokinases in the same family revealed that these cysteines are conserved only in PsGK and a closely related Antarctic *Pseudoalteromonas*‐derived glucokinase (PhGK) (Fig. 1). This suggests that the disulfide bond is not a random mutation, but rather a selectively maintained adaptation for sustaining enzymatic activity and structural stability under extreme cold conditions. In the Antarctic environment, where enzyme function is challenged by low temperatures and oxidative stress, the disulfide bond may have been selectively utilised to finely balance the trade‐off between catalytic efficiency and structural stability. Taken together, this study demonstrates that the disulfide bond in PsGK is not merely a thermal stabilising factor, but rather a highly evolved structural control element that simultaneously ensures local structural rigidity and catalytic function through a stability–flexibility balance. These insights significantly advance our understanding of structure–function relationships in cold‐adapted enzymes.

### Introduction of a PsGK‐like disulfide bond into EcGK

**Fig. 5:**
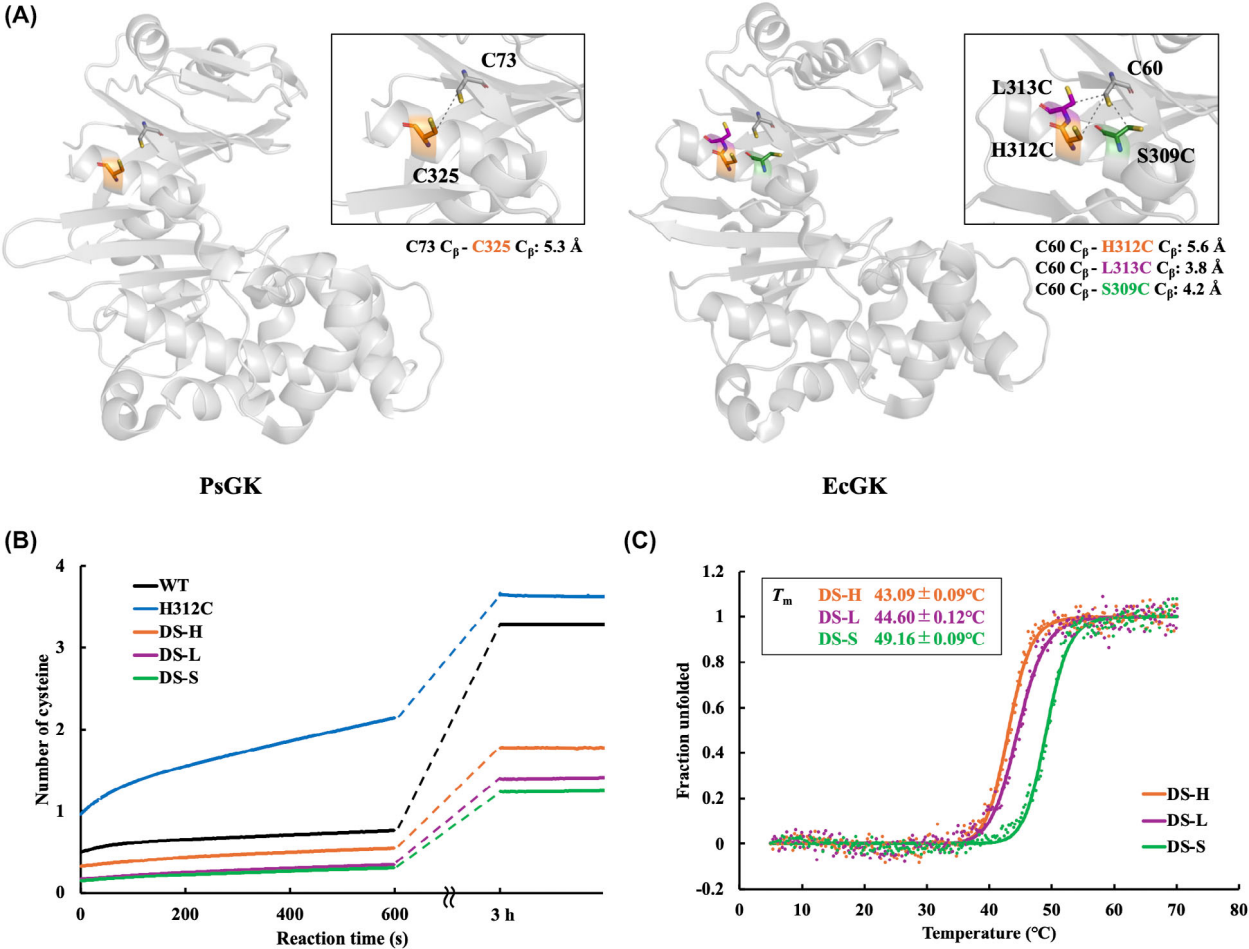
Introduction of disulfide bond into glucokinase from *Escherichia coli* (EcGK). (A) The position of cysteine residues (Cys73 and Cys325) involved in disulfide bonds in glucokinase from *Pseudoalteromonas* sp. AS‐131 (PsGK) (PDB ID: 3VPZ, left) and candidate sites of cysteine mutation for introduction of disulfide bonds in EcGK (PDB ID: 1Q18, right) are shown by sticks. Amino acid residues at positions at which is possible to form a disulfide bond with Cys60, Ser309 (green), His312 (orange), Leu313 (purple), are replaced with cysteine residues and highlighted in stick models. Cys73 (grey) and Cys325 (orange) in PsGK are located in homologous positions to Cys60 (grey) and His312 (orange), respectively. The distances between *C*_β_ of each cysteine residues are indicated. The image was prepared using PyMOL. (B) Comparison of the number of cysteines that reacted with 5′5′‐dithiobis(2‐nitrobenzonate) (DTNB) as a function of time in EcGK wild‐type (WT), H312C (blue), EcGK C20S C65S H312C (DS‐H, orange), EcGK C20S C65S L313C (DS‐L, purple), and EcGK C20S C65S S309C (DS‐S, green). 10 μm protein solution purified without reductant were reacted and monitored the absorbance at 412 nm for 10 min. After 3 h, the absorbance at 412 nm of the reaction mixture was measured. The number of accumulations was one. (C) Thermal melting curves of DS‐H (orange), DS‐L (purple), and DS‐S (green). The change in ellipticity at 222 nm was monitored as a function of temperature. The measurement temperature was 5–70 °C. The number of accumulations was one.

To examine the potential effect of introducing a PsGK‐like disulfide bond, which links the N and C termini, into the mesophilic EcGK, cysteine mutations were introduced at the corresponding positions (Fig. 5A), and the thermal stability of this variant (EcGK H312C) was evaluated. A summary of the EcGK variants used in this study is shown in Table 5. Ellman's method was used for EcGK variants to verify whether a disulfide bond was successfully formed. As shown in Fig. 5B, two cysteine residues reacted with DTNB immediately, and two more cysteine residues reacted slowly, suggesting that a disulfide bond was not formed in the EcGK H312C variant. According to the amino acid sequence and crystal structure of EcGK, the two cysteine residues (Cys20 and Cys65), which are not conserved in PsGK, are located near H312C, suggesting that these cysteine residues may interfere with the proper formation of a disulfide bond. Therefore, to eliminate this potential interference and more closely mimic the environment of PsGK, we prepared the EcGK C20S C65S H312C variant (EcGK DS‐H), which replaced these cysteine residues with serine residues in the EcGK H312C variant. The results of the DTNB experiment for EcGK DS‐H showed that almost two cysteine residues reacted with DTNB, indicating that no disulfide bond was formed because no cysteine residue would react if the disulfide bond was formed properly (Fig. 5B). In fact, the distance between *C*_β_ of cysteine residues of Cys60 and Cys312 was 5.6 Å, which was slightly farther than the desirable distance for forming a disulfide bond, < 5.5 Å [59], and was therefore considered to be the reason for the failure of disulfide bond formation. Suitable candidate pairs of cysteine residues capable of forming a disulfide bond between the N and C termini were predicted using the Disulfide by Design 2.0 program [66]. As candidates, two variants were selected in which the *C*_β_‐*C*_β_ distances between Cys60 and the introduced cysteine residues were 3.8 and 4.2 Å, respectively: EcGK C20S C65S L313C (EcGK DS‐L) and EcGK C20S C65S S309C (EcGK DS‐S) (Fig. 5A) [66]. DTNB experiments for DS‐L and DS‐S variants showed 1.4 and 1.3 cysteine residues reacted with DTNB, respectively, suggesting that partially formed disulfide bonds or limited accessibility of certain cysteine residues due to structural constraints might be responsible (Fig. 5B). Molecular dynamics (MD) simulations based on the crystal structure showed that, in DS‐L, the distance between the introduced cysteine sulfur (SG) atoms was 6.4 Å (Fig. S10a), suggesting that disulfide bond formation may be inefficient. These results suggest that structural constraints likely hinder DTNB access to the free cysteine residues. Examination of the structure obtained from MD simulations revealed that Cys60 was located within the interior of the enzyme. Furthermore, the solvent accessibility of the cysteine residues involved in disulfide bond formation in DS‐L was assessed using SASA analysis. The results showed that Cys60 was completely buried (SASA = 0 Å^2^), whereas Cys313 was partially solvent‐exposed, with a SASA value of 5.13 Å^2^, suggesting partial exposure to the solvent. These findings imply that DTNB was unable to access Cys60. In contrast, the SG‐SG distance in DS‐S was 3.8 Å, which is sufficiently short to allow disulfide bond formation (Fig. S10b). Furthermore, initiating the MD simulation from a structure containing a preformed disulfide bond showed that the bond remained stable throughout the trajectory (Fig. S10c), suggesting that DS‐S can accommodate disulfide bond formation without incurring significant structural strain.

**Table 5:** Summary of glucokinase from *Escherichia coli* (EcGK) variants used in this study. DS, EcGK C20S/C65S background; WT, wild‐type. DS‐H/S/L represent disulfide‐introduced variants at H312, S309, and L313, respectively. Only cysteine residues potentially involved in disulfide bond formation are listed. All variants retain Cys227, Cys230, and Cys238, which are not involved in disulfide engineering and thus are omitted from this column.

Abbreviation	Full name	Cysteine positions	Description
WT	EcGK (wild‐type)	C20, C60, C65	Native EcGK
H312C	EcGK H312C	C20, C60, C65, C312	Disulfide‐introduced variant at H312C
DS‐H	EcGK C20S C65S H312C	C60, C312	Disulfide‐introduced variant at H312C on DS background
DS‐L	EcGK C20S C65S L313C	C60, C313	Disulfide‐introduced variant at L313C on DS background
DS‐S	EcGK C20S C65S S309C	C60, C309	Disulfide‐introduced variant at S309C on DS background

To further verify this, the thermal stability of each variant was evaluated (Fig. 5C). The *T*_m_ values of DS‐H (43.09 ± 0.09 °C) significantly decreased by 7.9 °C compared to WT (51.00 ± 0.11 °C), consistent with the result of the DTNB assay indicating that DS‐H lacks disulfide bond formation. These results suggest that the DS‐H structure easily unfolds due to changes in the intra‐enzyme interactions caused by the C20S and C65S mutations. A comparison of the shape of the CD spectra of DS‐H with EcGK WT at 20 °C showed no significant differences, indicating that the mutations did not affect the overall folding of the enzyme (Fig. S11). The *T*_m_ value of DS‐L was 44.60 ± 0.12 °C, which was comparable to that of the DS‐H variant lacking a disulfide bond. In contrast, the DS‐S variant exhibited a *T*_m_ of 49.16 ± 0.09 °C, which is approximately 6.0 °C higher than the DS‐H variant, experimentally demonstrating that the PsGK‐like disulfide bond enhances thermal stability. Furthermore, principal component analysis (PCA) of the MD simulations revealed that the overall rigidity of the DS‐S variant was improved compared to the WT (Fig. S12).

Interestingly, the DS‐S variant exhibited higher specific activity than the WT (Fig. S13). In addition, *k*_cat_/*K*_m_ values of DS‐S exceeded those of the WT at 1 and 25 °C, but it decreased below WT at 40 °C (Table S2). At 1 °C, the catalytic activity of the WT enzymes is likely limited by reduced molecular dynamics, whereas the DS‐S variant may retain a catalytically favourable conformation through disulfide bond formation, similar to that observed in PsGK, thereby enhancing catalytic efficiency. At 25 °C, the structural constraint introduced by the disulfide bond may also stabilise a catalytically favourable conformation. In contrast, at 40 °C, excessive structural stabilisation resulting from the disulfide bond likely restricted the conformational flexibility required for catalysis, leading to reduced activity. These findings suggest that a certain degree of structural mobility is essential at higher temperatures and that this flexibility may have been compromised in the DS‐S variant. These results indicate that introducing a disulfide bond can improve not only the thermal stability of an enzyme but also catalytic performance when introduced at appropriate positions. However, these effects are temperature‐dependent; excessive structural constraint can impair catalytic activity at elevated temperatures. Therefore, in designing disulfide bonds, it is essential to balance structural stabilisation with the flexibility required for catalysis.

## Conclusions

In this study, we demonstrated that the Antarctic bacterial glucokinase PsGK possesses dual properties: it is a cold‐adapted enzyme that exhibits remarkably high thermal stability. PsGK maintained high catalytic efficiency even at 1 °C and exhibited a *T*_m_ of approximately 68.46 ± 1.07 °C, which is significantly higher than that of typical cold‐adapted enzymes, which are generally thermolabile. We identified a unique disulfide bond between Cys73 and Cys325 that bridges the N‐ and C‐terminal regions of PsGK, playing a crucial role in maintaining its thermal stability. Structural and mutational analyses revealed that this disulfide bond not only contributes to structural stabilisation but also facilitates the retention of catalytically favourable conformations under low‐temperature conditions. Thus, it appears to mediate a balance between structural rigidity and conformational flexibility—key features for enzymatic function in cold environments. To further investigate this mechanism, we introduced disulfide bonds into the mesophilic EcGK at positions homologous to those in PsGK. Our analyses showed that the efficiency of disulfide bond formation and the structural accessibility of the involved cysteine residues greatly influenced both thermal stability and catalytic activity in a temperature‐dependent manner. Notably, the DS‐S variant, which formed a disulfide bond successfully, exhibited enhanced stability and cold activity. However, excessive structural rigidity hindered activity at elevated temperatures.

In contrast to previous reports on cold‐active yet stable enzymes, PsGK represents the first example in which disulfide bond‐mediated stabilisation underlies both cold adaptation and high thermal resilience. These findings suggest that disulfide bonds can serve as sophisticated structural elements that locally stabilise key regions of the protein without compromising its catalytic flexibility. Our work therefore provides valuable insights into the structural adaptation strategies of extremozymes, highlighting the potential utility of disulfide engineering in designing enzymes for extreme environments.

## Materials and methods

### Cloning, expression and purification

It was assumed that the genes of *Pseudoalteromonas* sp. AS‐131 would be highly conserved with those of *Pseudoalteromonas haloplanktis* TAC125 since one of the DNA sequences, alkaline protease gene (*fpa*), from AS‐131 exhibited 99% identity to the *fpa* gene of TAC125. Subsequently, the nucleotide sequence of the glucokinase gene in AS‐131 was identified using the *phglk* sequence from TAC125. The protein encoded by this gene (UniProt ID: H7CHS4) was referred to as PsGK in this study. For the comparison with PsGK, the genome sequence of *Escherichia coli* K‐12 was obtained from GenBank, and the glucokinase gene (*glk*) was identified. The protein encoded by *glk* (UniProt ID: P0A6V8) was referred to as EcGK.

PsGK and EcGK DNAs were cloned into the expression vector pET‐16b by homologous recombination using a yeast‐based *in vivo* cloning method [67, 68]. These DNAs were amplified by PCR using the following primers: a forward primer, 5′‐TGTTTAACTTTAAGAAGGAGATATACC **ATG**AGCTTACACTCTTCTGCC‐3′, and a reverse primer, 3′‐CGCAGCTTCCTTTCGGGCTTTGTTAGCAGC **TTA**TTCCTGCTTACTATTATGCAAACAC‐5′ (NOTE: underlined and bold sequences represent homologues of the pET‐16b vector start and stop codon, respectively). The amplified DNA fragment and pET‐16b vector digested with *Bam*HI were mixed, and *Saccharomyces cerevisiae* YPH499 competent cells were transformed. Recombinant plasmid DNA was isolated from *S. cerevisiae* using the QIAprep Miniprep Kit (Qiagen, Germantown, MD, USA) [69].

The rescued plasmid was transformed into *Escherichia coli* (*E. coli*) DH5α for propagation [67]. The recombinant plasmid was transferred to *E. coli* DH5α [70]. The recombinant plasmid DNA isolated from *E. coli* DH5α was transformed into *E. coli* BL21 (DE3) for expression. Similarly, the gene encoding EcGK was amplified by PCR from the genomic DNA of *E. coli* K‐12 obtained from GenBank and cloned into the expression vector pET‐16b. In addition, PsGK C156S, EcGK C20S, EcGK C60S and EcGK C65S, for the identification of cysteines exposed to solution, and PsGK C325S, EcGK H312C, EcGK C20S C65S H312C, EcGK C20S C65S L313C and EcGK C20S C65S S309C, for CD spectroscopy, were synthesised by GenScript Biotech and cloned into the expression vector pET‐16b digested with *Nco*I and *Bam*HI.

*E. coli* BL21 (DE3) was purchased from Funakoshi and transformed with the expression vector, inoculated into LB medium containing 50 μg·mL^−1^ of ampicillin, and grown at 37 °C until OD_660nm_ was 0.6–1.0. Isopropyl‐1‐thio‐β‐*d*‐galactopyranoside (IPTG) was added to the culture medium (a final concentration of 1.0 mm), and protein expression was induced at 20 °C for 24 h. All conditions for culturing were optimised to achieve high expression of GK. The harvested cells were suspended in 20 mm Tris–HCl buffer (pH 7.6) containing 10 mm MgCl_2_, followed by the addition of dithiothreitol (DTT), phenylmethylsulfonyl fluoride, and lysozyme added to the cell suspension (final concentrations of 1.0 mm, 1.0 mm and 0.08%, respectively), and incubated on ice for approximately 20 min. Sodium deoxycholate was added at a final concentration of 0.2% and stored at −80 °C. The cell suspension was then disrupted by freezing and thawing. Streptomycin was added to the suspension at a final concentration of 2.0% to remove nucleic acids. The suspension was gently stirred and centrifuged at 10 000 rpm for 20 min. The supernatant was collected and subjected to ammonium sulfate fractionation. The active fractions of 20–50% and 30–60% ammonium sulfate saturation for PsGK and EcGK, respectively, were collected and dialysed with 20 mm Tris–HCl buffer (pH 7.6) containing 10 mm MgCl_2_ and 5 mm β‐mercaptoethanol. The dialysed samples were loaded onto the anion‐exchange chromatography using HiTrap Q HP (Cytiva, Uppsala, Sweden), and the bound protein was eluted with a linear gradient of KCl (0–300 mm in 20 mm Tris–HCl buffer (pH 7.6) containing 10 mm MgCl_2_ and 5 mm β‐mercaptoethanol). The expression and purity of PsGK and EcGK were assessed by SDS/PAGE [71]. The molecular weights of the purified PsGK WT and EcGK WT were estimated by gel filtration using a HiLoad 16/60 Superdex 200 prep grade column (Cytiva). Protein concentration was determined by measuring the absorbance at 280 nm (*ε*_280nm_ = 21.4 mm·cm^−1^ and 28.4 mm^‐1^ ·cm^−1^ for PsGK and EcGK, respectively).

### Activity assay

The glucokinase activity was measured using a two‐step reaction. For the first step, a reaction mixture containing 10 μL of enzyme and 290 μL of substrate (5 mm glucose and 2 mm ATP) in 20 mm Tris–HCl buffer (pH 7.6) containing 10 mm MgCl_2_ and 1 mm DTT was incubated for 5 min at various temperatures (1–80 °C). The reaction was stopped by the addition of 15 μL of 50% (w/w) trichloroacetic acid and neutralised by the addition of 30 μL of 2 m Tris. In the second step, a reaction mixture from the first step and 50 μm d‐glucose 6‐phosphate dehydrogenase (G6PD) in 20 mm Tris–HCl buffer (pH 7.6) containing 10 mm MgCl_2_ and 1.2 mm NADP^+^ was incubated for 20–30 min at room temperature. The amount of glucose 6‐phosphate produced by glucokinase was estimated by measuring the absorbance of NADPH, the product of the second step, at 340 nm using a microplate reader (Thermo Scientific, Waltham, MA, USA). One unit of glucokinase activity was defined as the enzymatic activity required to phosphorylate 1.0 μmol of glucose to 1.0 μmol glucose 6‐phosphate per minute under described conditions.

### Thermal stability

The thermal stability of the glucokinases was determined by measuring residual activity and CD spectroscopy. Residual activities were determined by the following two experiments. In the first experiment, the enzyme solutions were heated at various temperatures ranging from 45 to 70 °C for up to 60 min. At each temperature, samples were taken every 10 min and rapidly cooled on ice. Residual activity was determined under the optimum reaction conditions for each enzyme, with the initial activity at zero time defined as 100%. The *k*_d_ was determined from the slope of a plot of the log of residual activity against time. In the second experiment, the residual activities were measured at optimum conditions after pre‐incubation at various temperatures for 10 min in 20 mm Tris–HCl (pH 7.6) buffer containing 10 mm MgCl_2_, evaluated with the specific activity at optimum conditions as 100%. To estimate the *T*_50_, the residual activity measured over time was fitted to a logistic regression model.

CD measurements were taken using a Jasco J‐1500 spectropolarimeter at the Institute for Molecular Science, Okazaki, equipped with a Peltier cell holder and a PTC‐510 temperature controller using a quartz cell with a 0.1 mm path length (Jasco, Hachioji, Tokyo, Japan). The number of accumulations was one. To assess the secondary structure, CD spectra were collected at 20 °C in the wavelength range of 200–260 nm. The measurement parameters were set as follows: 0.1 nm data pitch, 20 nm·min^−1^ scanning speed, 1 nm band width, 1 s response time and 100 mdeg sensitivity. The enzymes were prepared in a 10 mm potassium phosphate buffer (pH 7.6) at a concentration of 5 μm. For thermal denaturation analysis, the ellipticity at 222 nm was monitored while gradually increasing the temperature from 5 to 90 °C, with data recorded at 0.2 °C intervals under continuous temperature control by the Peltier device [72]. The unfolding curves were analysed using a sigmoidal curve function, according to Rünzler *et al*. [73] (Eqn 1):

(1) $$\theta_{T}=\left[\frac{\left(m_{D}\times T-b_{D}\right)-\left(m_{N}\times T-b_{N}\right)}{1+{\left(T/T_{m}\right)}^{m^{T}}}\right]+m_{N}\times T-b_{N}$$

where *θ*_*T*_ is the ellipticity at temperature *T*, *m* ^T^ is the slope of the curve within the transition region, and the inflexion point of the curve represents the melting temperature *T*_m_. The *T*_m_ values were calculated from these thermal melting curves. The fitting errors of *T*_m_ were used as the standard errors of the measurements. At each temperature *b*_*N*_ and *b*_*D*_ can be extrapolated from the pre‐ and post‐transition baselines, (*m*_N_ × *T* − *b*_N_) and (*m*_*D*_ × *T* − *b*_D_), respectively. The fraction of unfolded proteins was calculated by subtracting the baseline values (Eqn 2).

(2) $$\mathrm{fU}=\frac{\theta_{T}-\theta_{N}}{\theta_{U}-\theta_{N}}=\frac{\theta_{T}-\left(m_{N}\times T-b_{N}\right)}{\left(m_{D}\times T-b_{D}\right)-\left(m_{N}\times T-b_{N}\right)}$$

*T*_gap_ was defined as the temperature difference between *T*_m_ and *T*_opt_ and calculated from the following equation (Eqn 3) [44].

(3) $$T_{\mathrm{gap}}=T_{m}-T_{\mathrm{opt}}$$

### Kinetic parameter

*K*_m_ and *k*_*c*at_ of PsGK and EcGK were measured at 1, 25 and 40 °C in 20 mm Tris–HCl (pH 7.6) buffer containing 10 mm MgCl_2_, 5 mm glucose, 2 mm ATP, 1 mm DTT, 1.2 mm nicotinamide adenine dinucleotide phosphate (NADP^+^) and 50 μm G6PD. The concentrations of glucose and ATP varied from 0.17 to 1.0 mm. *K*_m_ and *k*_*c*at_ for each substrate were determined by steady‐state experiments performed at 1, 25 and 40 °C in assay buffer containing 20 mm Tris–HCl (pH 7.6), 10 mm MgCl_2_, 1 mm DTT, 1.2 mm NADP^+^, 50 μm G6PD and various concentrations of glucose and ATP, using Lineweaver–Burk plots based on Michaelis–Menten enzyme kinetics [74]. The values represent mean values ± standard deviation of three independent experiments in Table 1.

### Thermodynamic parameter

The *E*_a_ of the reaction catalysed by PsGK and EcGK was calculated by measuring the slope of the Arrhenius plot, which was made based on the *k*_cat_ values at 1–40 °C, employing the following equation (Eqn 4):

(4) $$\ln k_{\mathrm{cat}}=\ln A-E_{a}/\mathrm{RT}$$

*k*_cat_ values used in the Arrhenius plot were calculated based on the reaction rates measured at given temperatures. The thermodynamic activation parameters of PsGK and EcGK at 25 °C were calculated using Equations [75] (Eqns 5, 6 and 7).

(5) $$\Delta G^{\#}=\Delta H^{\#}-T\Delta S^{\#}$$

(6) $$\Delta H^{\#}=E_{a}-\mathrm{RT}$$

(7) $$\Delta S^{\#}=2.303R\left(\log k_{\mathrm{cat}}-10.753-\log T+E_{a}/2.303\mathrm{RT}\right)$$

### DTNB method

Ellman's reagent, DTNB, has the ability to bind to the free thiol groups by cleaving its own disulfide bond and releasing 5‐mercapto‐2‐nitrobenzoic acid (TNB) [76]. Spontaneously, TNB is ionised to the TNB^2−^ dianion in water at neutral and alkaline pH. As DTNB is reacted with the free thiol group of cysteine at a one‐to‐one ratio, the concentration of free thiol groups can be quantified by measuring the absorbance of TNB^2−^ [77, 78]. Furthermore, DTNB reacts slowly or does not react with free cysteine residues inside the enzyme depending on the degree of structural flexibility [79]. To avoid the reaction of DTNB with the reductant, β‐mercaptoethanol in the samples was removed using a desalting spin column (APRO Science group, Myozai, Tokushima, Japan). DTNB was dissolved in 10 mm phosphate buffer (pH 6.5) to a final concentration of 4 mm. The mixing ratio of enzyme, 0.1 m Tris–HCl buffer (pH 8.0) containing 1 mm ethylenediaminetetraacetic acid, was adjusted by the number of expected free cysteine residues so that the absorbance at 412 nm of TNB^2−^ (*λ*_max_ = 412 nm, *ε* = 14 150 m ^−1^·cm^−1^) was approximately 0.2 per one cysteine residue. DTNB was added at a molar amount 20‐fold greater than that of the enzyme. The reaction mixture was initiated to react for 1 min and then started to record the absorbance at 412 nm for 5–10 min. After 3–12 h, the absorbance was measured at 412 nm. The number of accumulations was one for each measurement.

### Molecular dynamics

MD simulations were performed using the AMBER16 program package [80]. The protein structure was obtained from the Protein Data Bank (PDB ID: 1Q18), and specific amino acid residues were replaced with cysteines (S309C and L313C) to allow disulfide bond formation. The disulfide bond was explicitly defined using the bond command in LEaP. The ff14SB all‐atom force field was applied [81]. Water was modelled by the TIP3P potential, and a protein molecule was surrounded by a periodic octahedral box of TIP3P water molecules. Na^+^ counterions were added to neutralise the system. All Lennard–Jones interactions were cut off at a distance of 10 Å, and long‐range electrostatic interactions were calculated using a particle mesh Ewald method [82]. The MD simulations were set up using the following protocol. First, 1000 steps of energy minimisation were performed to remove close van der Waals contacts present in the initial structure and to allow the formation of hydrogen bonds between solvent molecules and the protein. In this stage, the protein was kept fixed, and only the positions of water molecules were minimised. In the second step, an unrestrained energy minimisation for 2500 steps, in which the entire system was minimised. As the third step, the system was then gradually heated to 300 K over 20 ps under constant volume conditions. The temperature was controlled using Langevin dynamics with a collision frequency of 1.0 ps^−1^ [83]. To constrain bonds involving hydrogen atoms, the SHAKE algorithm was employed, and the time integration step was set to 2 fs [84]. After heating, the system was simulated for 50 ns under constant temperature (300 K) and pressure (1 atm) conditions, using a time step of 2 fs. The distance between the SG of the cysteine residues forming the disulfide bond was extracted from all simulation frames and analysed to evaluate its time‐dependent variation.

## Author contributions

AY performed the main experiments and analysed the data. YK and FH performed the Ellman experiments. RA performed part of the activity assay. AO and TO built the vector of PsGK WT and EcGK WT. AY, ST and MU performed MD simulations. NS and KW contributed to the interpretation of data and designed part of this work. MH designed the study. AY and MH wrote the manuscript. All authors read and approved the manuscript.

## Conflict of interest

The authors declare no conflict of interest.

## Supporting information


**Table S1.** Thermal deactivation constants (*k*_d_) (min^−1^).
**Table S2.** Kinetic constants of glucokinase from *Pseudoalteromonas* sp. AS‐131 (PsGK) C325S and glucokinase from *Escherichia coli* (EcGK) C20S C65S S309C (DS‐S).
**Fig. S1.** Analytical anion exchange chromatography of (a) glucokinase from *Pseudoalteromonas* sp. AS‐131 (PsGK) wild‐type (WT) and (b) glucokinase from *Escherichia coli* (EcGK) WT using HiTrap Q HP.
**Fig. S2.** Sodium dodecyl sulfate‐polyacrylamide gel electrophoresis (SDS/PAGE) analysis of glucokinase from *Pseudoalteromonas* sp. AS‐131 (PsGK) wild‐type (WT) and glucokinase from *Escherichia coli* (EcGK) WT.
**Fig. S3.** Analytical gel filtration of glucokinase from *Pseudoalteromonas* sp. AS‐131 (PsGK) wild‐type (WT) and glucokinase from *Escherichia coli* (EcGK) WT.
**Fig. S4.** Plot of relative activity of glucokinase from *Pseudoalteromonas* sp. AS‐131 (PsGK) wild‐type (WT) and glucokinase from *Escherichia coli* (EcGK) WT as a function of temperature.
**Fig. S5.** Arrhenius plot of the substrate turnover (*k*_cat_) of glucokinase from *Pseudoalteromonas* sp. AS‐131 (PsGK) and glucokinase from *Escherichia coli* (EcGK).
**Fig. S6.** Thermal stability of glucokinase from *Pseudoalteromonas* sp. AS‐131 (PsGK) wild‐type (WT) and glucokinase from *Escherichia coli* (EcGK) WT.
**Fig. S7.** Residual activities of glucokinase from *Pseudoalteromonas* sp. AS‐131 (PsGK) wild‐type (WT) and glucokinase from *Escherichia coli* (EcGK) WT.
**Fig. S8.** Disulfide bond and hydrogen bonds.
**Fig. S9.** Plot of specific activity and relative activity of glucokinase from *Pseudoalteromonas* sp. AS‐131 (PsGK) wild‐type (WT) and PsGK C325S as a function of temperature.
**Fig. S10.** The distance between cysteine sulfur (SG) atoms.
**Fig. S11.** Circular dichroism (CD) spectrum of glucokinase from *Escherichia coli* (EcGK) wild‐type (WT), EcGK C20S C65S H312C (DS‐H), EcGK C20S C65S L313C (DS‐L), and EcGK C20S C65S S309C (DS‐S) at 20 °C.
**Fig. S12.** Principal component analysis (PCA) projection of glucokinase from *Escherichia coli* (EcGK) wild‐type (WT) and EcGK C20S C65S S309C (DS‐S).
**Fig. S13.** Plot of specific activity of glucokinase from *Escherichia coli* (EcGK) wild‐type (WT) and EcGK C20S C65S S309C (DS‐S) as a function of temperature.

## Data Availability

All data in this manuscript were available at Dryad (https://doi.org/10.5061/dryad.ncjsxkt6b).
