# Supplementary material for: Characterisation of a cold‐adapted, thermostable glucokinase from psychrophilic Pseudoalteromonas sp. AS‐131 reveals how the enzyme achieves high thermal stability without loss of cold adaptation
Source: FEBS J. 2025 Dec 15;293(9):2617–36. doi: 10.1111/febs.70367 (PMC13147314; doi:10.1111/febs.70367)

Characterisation of a cold-adapted, thermostable glucokinase from psychrophilic *Pseudoalteromonas* sp. AS-131 reveals how the enzyme achieves high thermal stability without loss of cold adaptation

Akane Yato[1], Yuki Kato[2], Fuyuko Hayashi[2], Rio Asaka[2], Atsuko Ogawa[2], Tokuro Oda[2], Sayaka Tsuji[1], Masashi Unno[3], Nobuaki Soh[1,2], Keiichi Watanabe[2], Masaki Horitani[1,2]*

[1]The United Graduate School of Agricultural Sciences, Kagoshima University, 1-21-24 Korimoto, Kagoshima, Kagoshima 890-0065, Japan

[2]Department of Applied Biochemistry and Food Science, Faculty of Agriculture, Saga University, 1 Honjyo-machi, Saga, Saga 840-8502, Japan

[3]Department of Chemistry and Applied Chemistry, Faculty of Science and Engineering, Saga University, 1 Honjo-machi, Saga, Saga 840-8502, Japan

*Correspondence: horitani@cc.saga-u.ac.jp; +81-952-28-8782

This PDF file includes:

Table S1 to 2 and Figs. S1 to S13

Table S1 Thermal deactivation constants (k_d) (min^{-1}).

enzyme[a]	45°C	50°C	55°C	60°C	65°C	70°C
PsGK	n.d.	n.d.	1.3×10^{-3}	5.4×10^{-3}	4.1×10^{-2}	1.8×10^{-1}
EcGK	5.6×10^{-3}	5.5×10^{-2}	3.5×10^{-1}	n.d.	n.d.	n.d.

[a]PsGK: glucokinase from *Pseudoalteromonas* sp. AS-131, EcGK: glucokinase from *Escherichia coli*

Table S2 Kinetic constants of glucokinase from *Pseudoalteromonas* sp. AS-131 (PsGK) C325S and glucokinase from *Escherichia coli* (EcGK) C20S C65S S309C (DS-S).

Kinetic constants	PsGK C325S			EcGK DS-S		
	1°C	25°C	40°C	1°C	25°C	40°C
$K_m^{glucose}$ (mM)	0.10 ± 0.03	0.21 ± 0.03	0.29 ± 0.17	0.83 ± 0.12	0.37 ± 0.03	0.91 ± 0.13
K_m^{ATP} (mM)	0.52 ± 0.04	0.96 ± 0.09	1.86 ± 0.25	0.42 ± 0.03	0.78 ± 0.15	1.46 ± 0.19
k_{cat} (s^{-1})	15.74 ± 0.46	48.57 ± 3.23	100.42 ± 1.85	23.45 ± 2.87	42.94 ± 2.28	63.08 ± 6.89
k_{cat}/K_m ($mM^{-1}s^{-1}$)	159.88 ± 33.79	237.85 ± 18.73	247.99 ± 1.74	28.29 ± 0.74	116.65 ± 3.92	69.12 ± 2.66

Fig. S1 Analytical anion exchange chromatography of (a) glucokinase from *Pseudoalteromonas* sp. AS-131 (PsGK) wild type (WT) and (b) glucokinase from *Escherichia coli* (EcGK) WT using HiTrap Q HP.

Analytical anion exchange chromatography of (a) PsGK WT and (b) EcGK WT was performed using HiTrap Q HP. The activities of PsGK and EcGK were detected from the chromatographic peak in the range of 150 to 250 mM and 50 to 150 mM KCl concentration on the liner gradient of KCl, respectively.

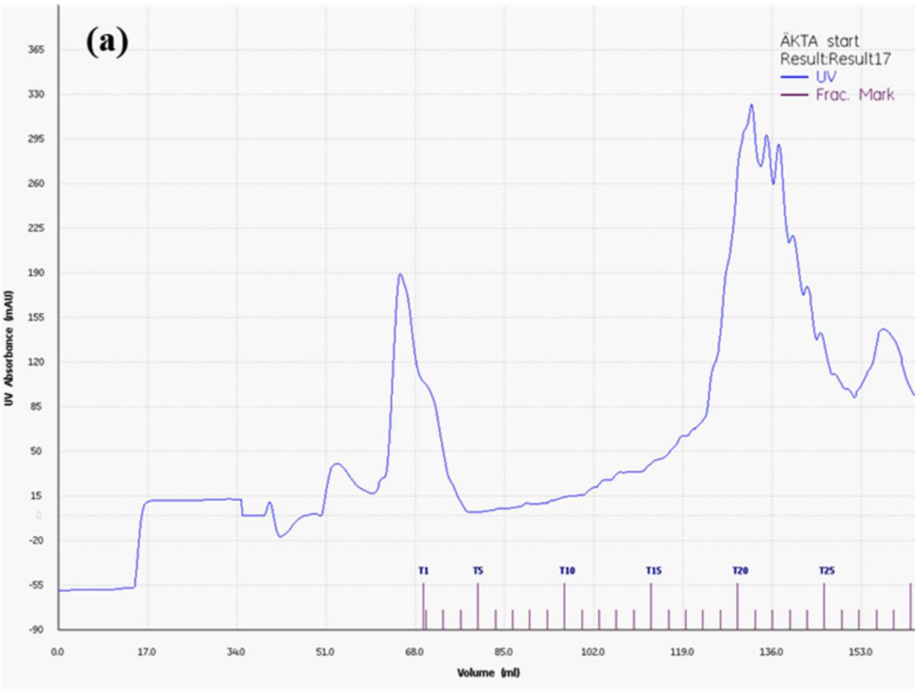

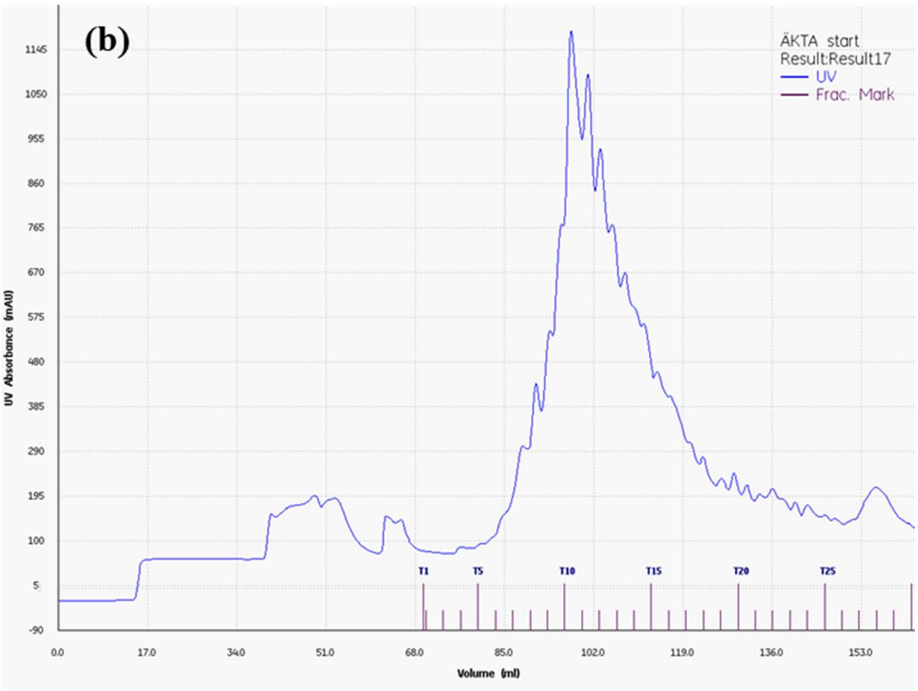

Fig. S2 Sodium dodecyl sulfate-polyacrylamide gel electrophoresis (SDS-PAGE) analysis of glucokinase from *Pseudoalteromonas* sp. AS-131 (PsGK) wild type (WT) and glucokinase from *Escherichia coli* (EcGK) WT.

SDS-PAGE analysis of the different steps in the expression and purification of (a) PsGK WT and (b) EcGK WT. Lane 1 and 4, precipitate obtained from cell extract of *E. coli* BL21(DE3)/PsGK and EcGK, respectively grown in LB at 20℃ for 24 h after expression was induced by IPTG addition; lane 2 and 5, protein precipitated at 20 to 50% and 30 to 60% saturated ammonium sulfate, respectively; lane 3 and 6, preparations after anion-exchange chromatography (HiTrap Q HP). The positions of PsGK and EcGK are indicated by arrows.

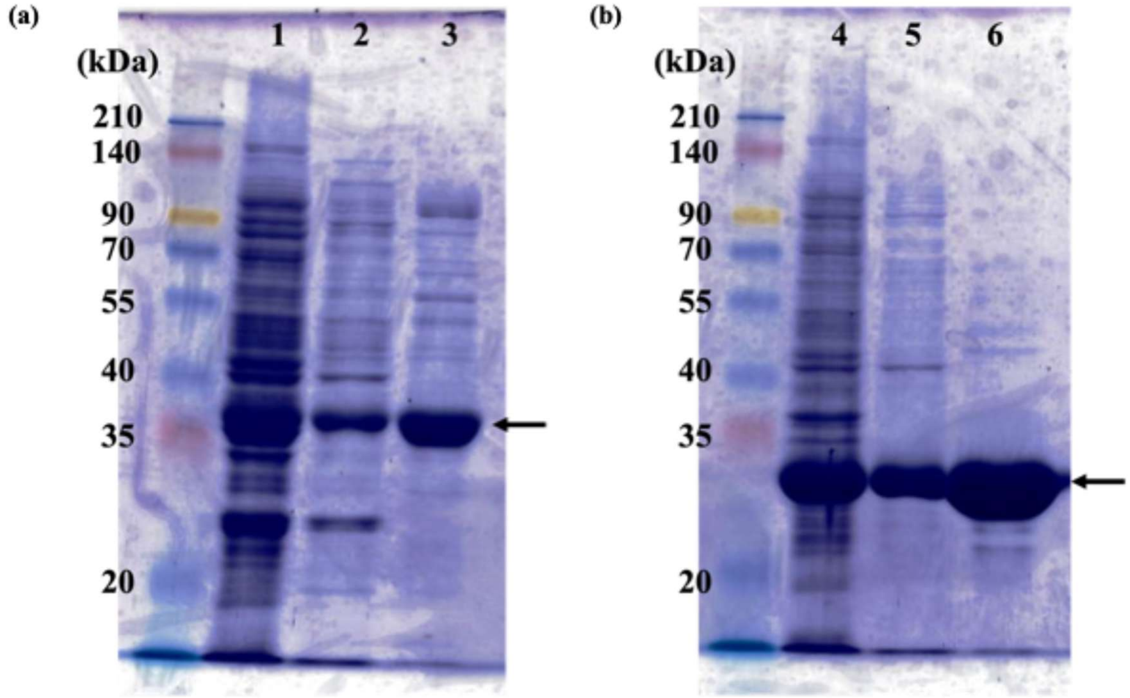

Fig. S3 Analytical gel filtration of glucokinase from *Pseudoalteromonas* sp. AS-131 (PsGK) wild type (WT) and glucokinase from *Escherichia coli* (EcGK) WT.

Analytical gel filtration of (a) PsGK WT and (b) EcGK WT using Superdex75 prep grade. These eluted positions were indicated dimer.

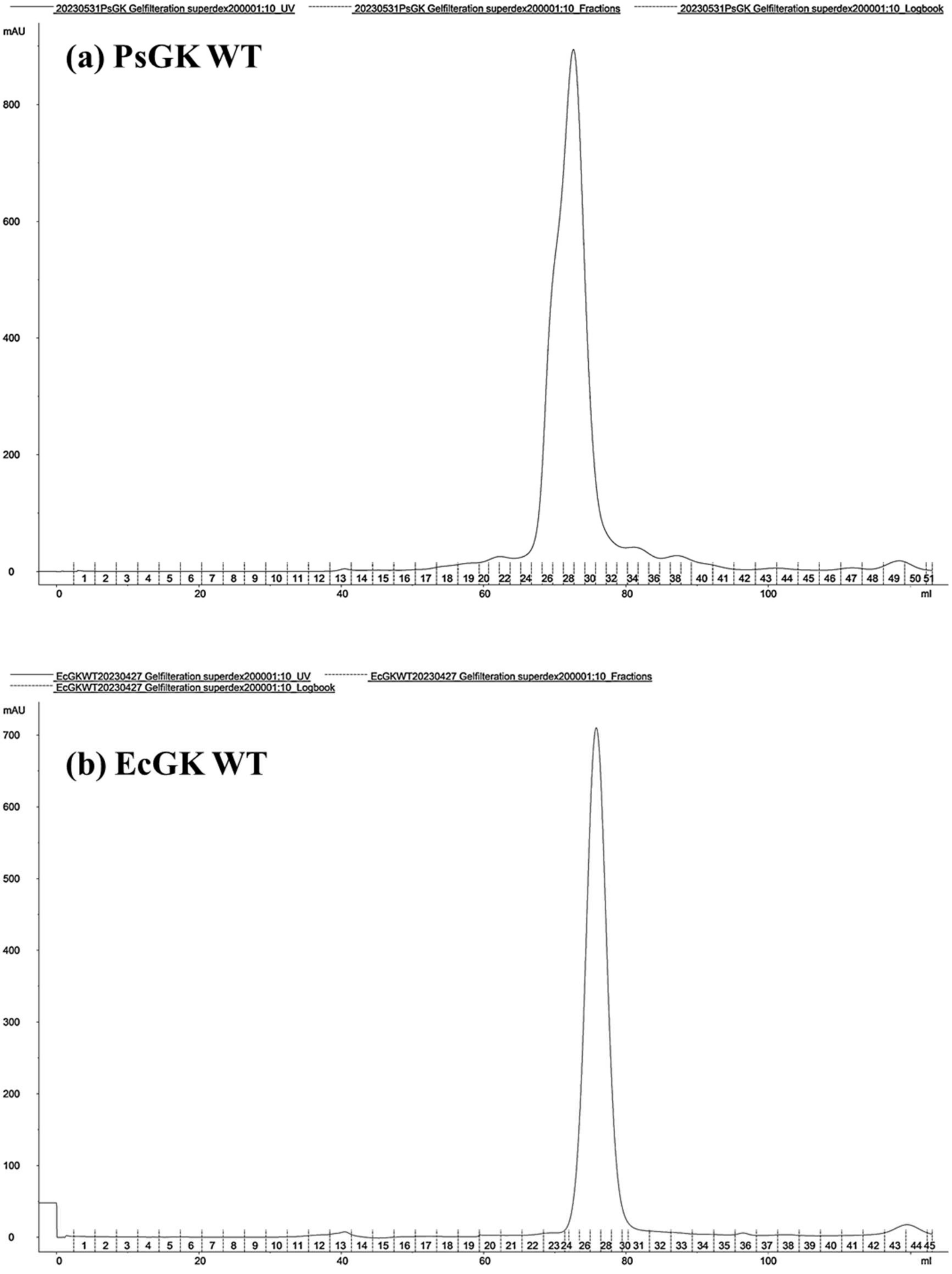

Fig. S4 Plot of relative activity of glucokinase from *Pseudoalteromonas* sp. AS-131 (PsGK) wild type (WT) and glucokinase from *Escherichia coli* (EcGK) WT as a function of temperature.
The relative values of specific activity at various temperatures compared with the activity at the optimum temperature for each enzyme, PsGK WT and EcGK WT. Both data were reproduced from the data shown in **Fig. 2(a)** for comparison of relative activity.

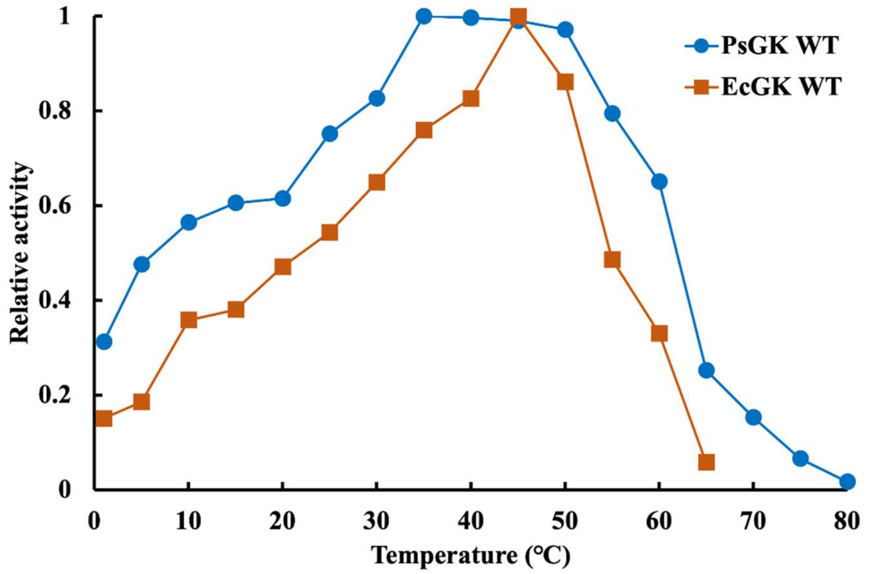

Fig. S5 Arrhenius plot of the substrate turnover (k_{cat}) of glucokinase from *Pseudoalteromonas* sp. AS-131 (PsGK) and glucokinase from *Escherichia coli* (EcGK).

Activation energy (E_a) was calculated from the slope of each plot. E_a: PsGK, 23.56 ± 1.37 kJ/mol; EcGK, 31.20 ± 0.83 kJ/mol. The values represent mean values ± standard deviation of three independent experiments (n = 3).

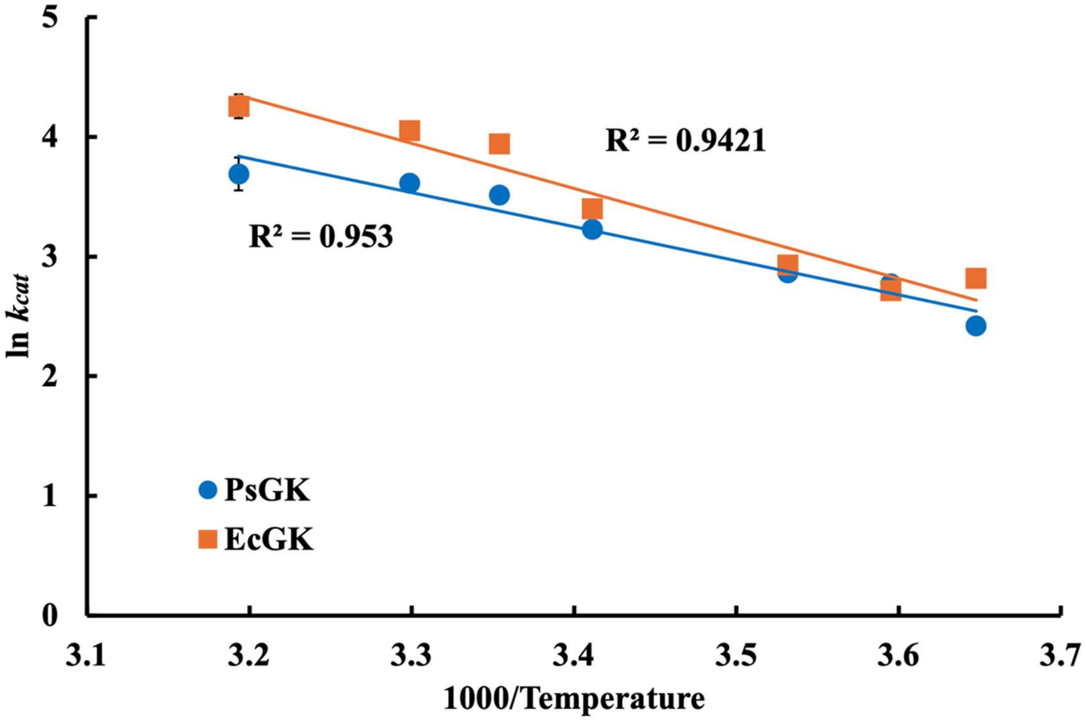

Fig. S6 Thermal stability of glucokinase from *Pseudoalteromonas* sp. AS-131 (PsGK) wild type (WT) and glucokinase from *Escherichia coli* (EcGK) WT.

The data were replotted from the data shown in **Fig. 2(b)** as a function of residual activity. The values represent mean values ± standard deviation of three independent experiments (n = 3).

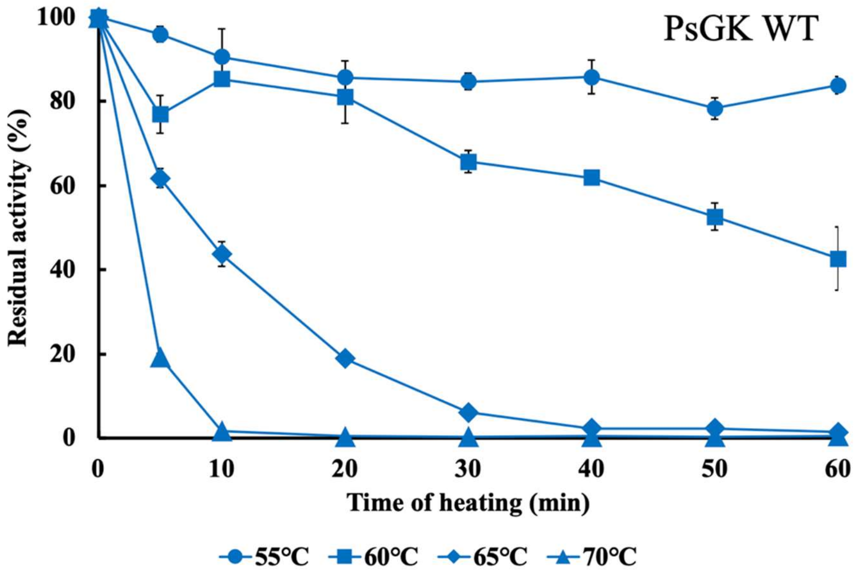

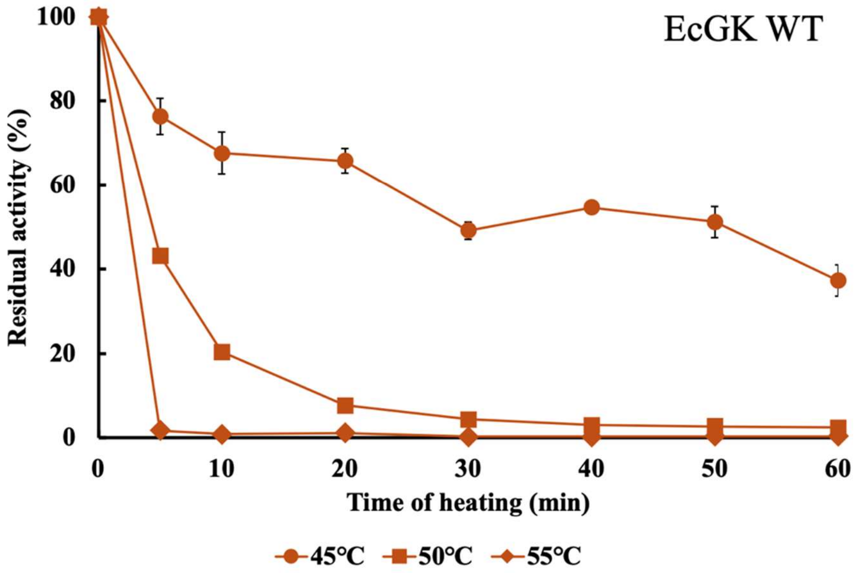

Fig. S7 Residual activities of glucokinase from *Pseudoalteromonas* sp. AS-131 (PsGK) wild type (WT) and glucokinase from *Escherichia coli* (EcGK) WT.

(a) Thermal stability examined by residual activity. The residual activities were measured at the optimum conditions after pre-incubation at various temperatures for 10 min in 20 mM Tris-HCl (pH 7.6), 10 mM $MgCl_2$. The values represent mean values ± standard deviation of three independent experiments (n = 3). Time-course of residual activity and logistic regression fitting of (b) PsGK WT and (c) EcGK WT. Experimental data (dots) were fitted to a logistic function (solid line) to estimate half-inactivation time (T_{50}), indicated by the blue dashed line. Experimental data were replotted from **Fig. S7(a)**.

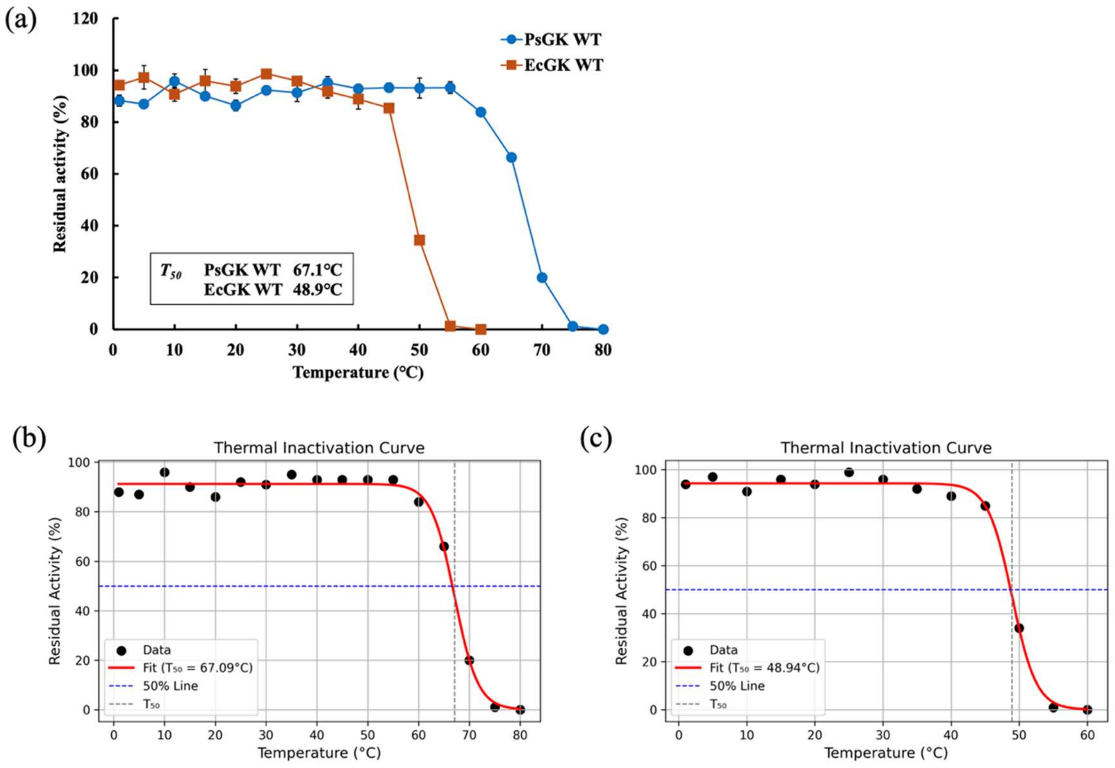

Fig. S8 Disulfide bond and hydrogen bonds.

(a) Cys73-Cys325 disulfide bond in glucokinase from *Pseudoalteromonas* sp. AS-131 (PsGK) wild type (WT) . N- and C-terminus are indicated. (b) β-sheets in small domain of PsGK WT (blue) and glucokinase from *Escherichia coli* (EcGK) WT (green). Hydrogen bonds are showed by dashed line colored magenta and the number of hydrogen bonds are indicated. The images were obtained using PyMOL.

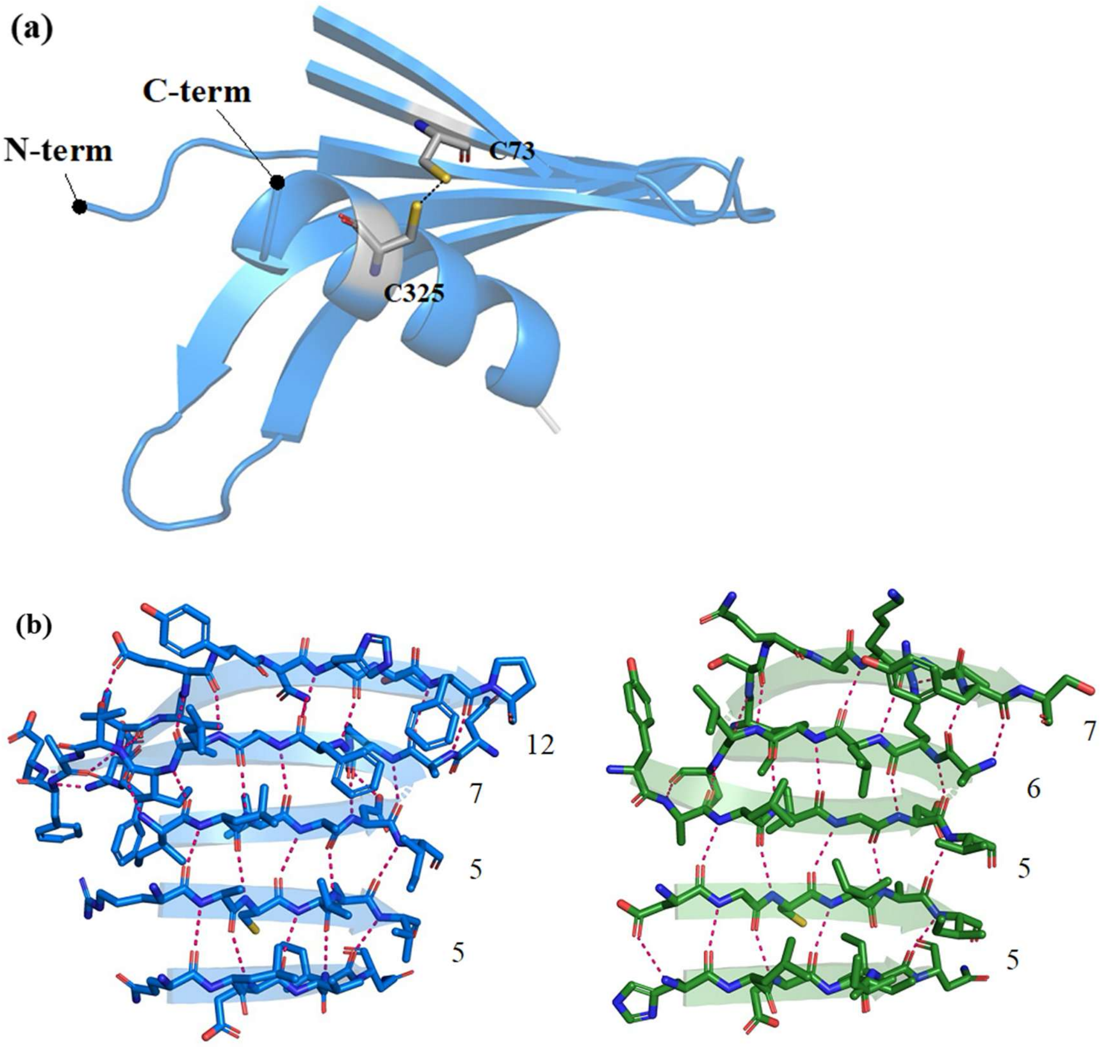

Fig. S9 Plot of specific activity and relative activity of glucokinase from *Pseudoalteromonas* sp. AS-131 (PsGK) wild type (WT) and PsGK C325S as a function of temperature.

The values represent mean values ± standard deviation of three independent experiments (n = 3). The relative values of specific activity at various temperatures compared with the activity at the optimum temperature for each enzyme. PsGK WT was replotted from the data shown in **Fig. 2(a)** for comparison.

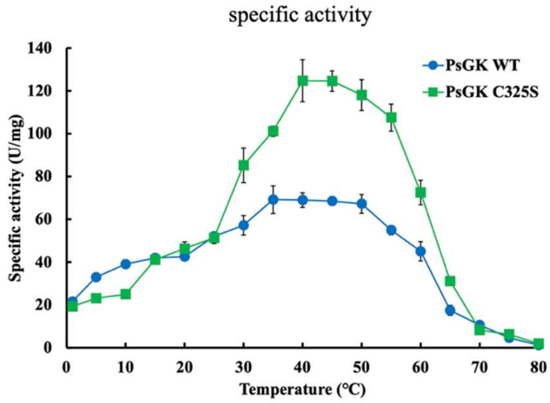

Fig. S10 The distance between cysteine sulphur (SG) atoms.

The distance between SG atoms in (a) glucokinase from *Escherichia coli* (EcGK) C20S C65S L313C (DS-L), (b) EcGK C20S C65S S309C (DS-S), and (c) DS-S containing pre-formed disulfide bond as a function of time, predicted by molecular dynamics (MD) simulations based on the crystal structure.

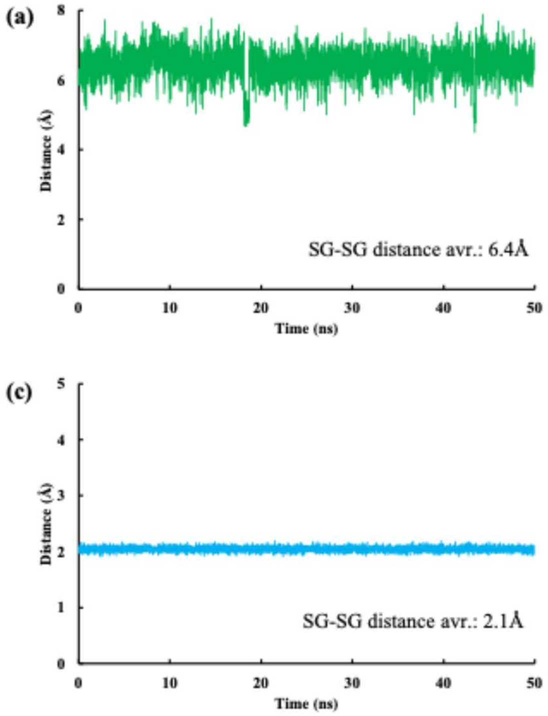

Fig. S11 Circular dichroism (CD) spectrum of glucokinase from *Escherichia coli* (EcGK) wild type (WT), EcGK C20S C65S H312C (DS-H), EcGK C20S C65S L313C (DS-L), and EcGK C20S C65S S309C (DS-S) at 20°C.

The measurement wavelength was 200-260 nm. The number of accumulations were four.

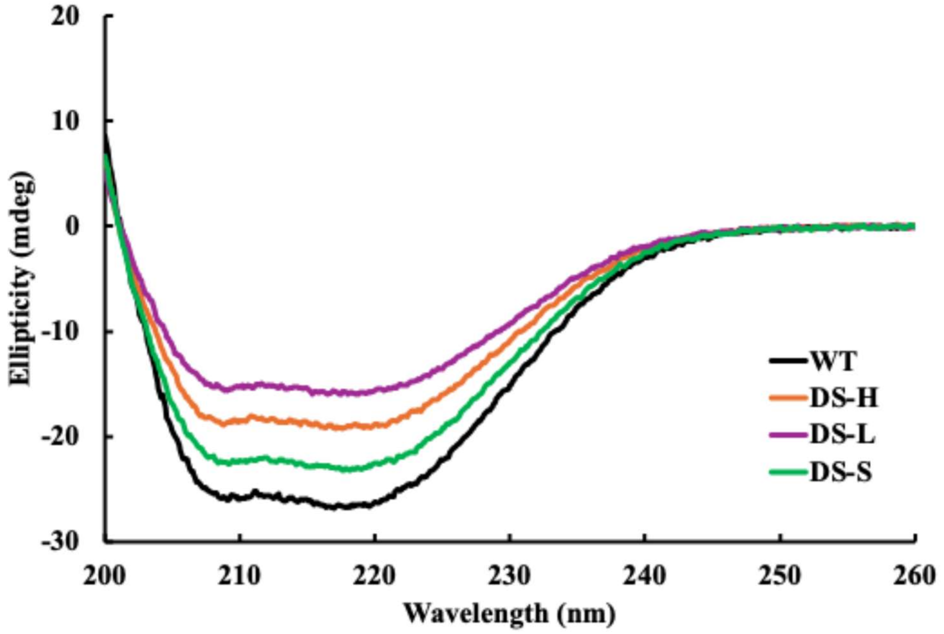

Fig. S12 Principal component analysis (PCA) projection of glucokinase from *Escherichia coli* (EcGK) wild type (WT) and EcGK C20S C65S S309C (DS-S).

PCA projection of EcGK WT and DS-S were shown in blue and red, respectively.

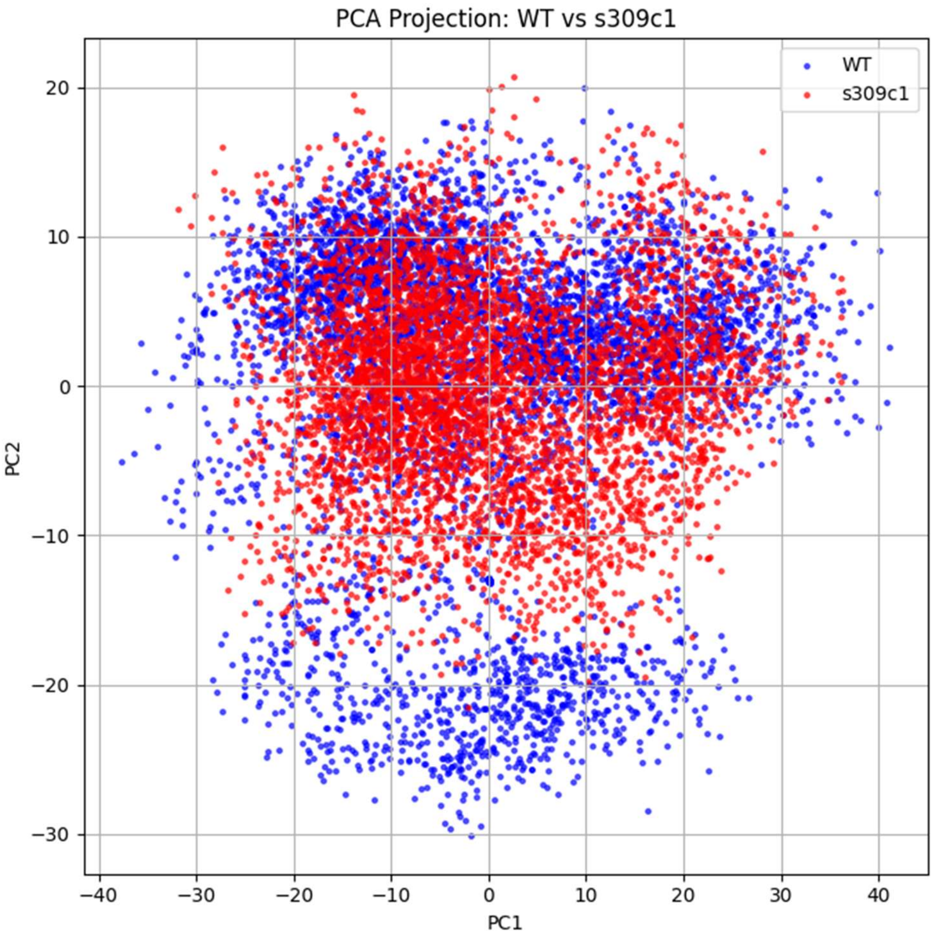

Fig. S13 Plot of specific activity of glucokinase from *Escherichia coli* (EcGK) wild type (WT) and EcGK C20S C65S S309C (DS-S) as a function of temperature.

The values represent mean values ± standard deviation of three independent experiments (n = 3). EcGK WT was replotted from the data shown in **Fig. 2(a)** for comparison.

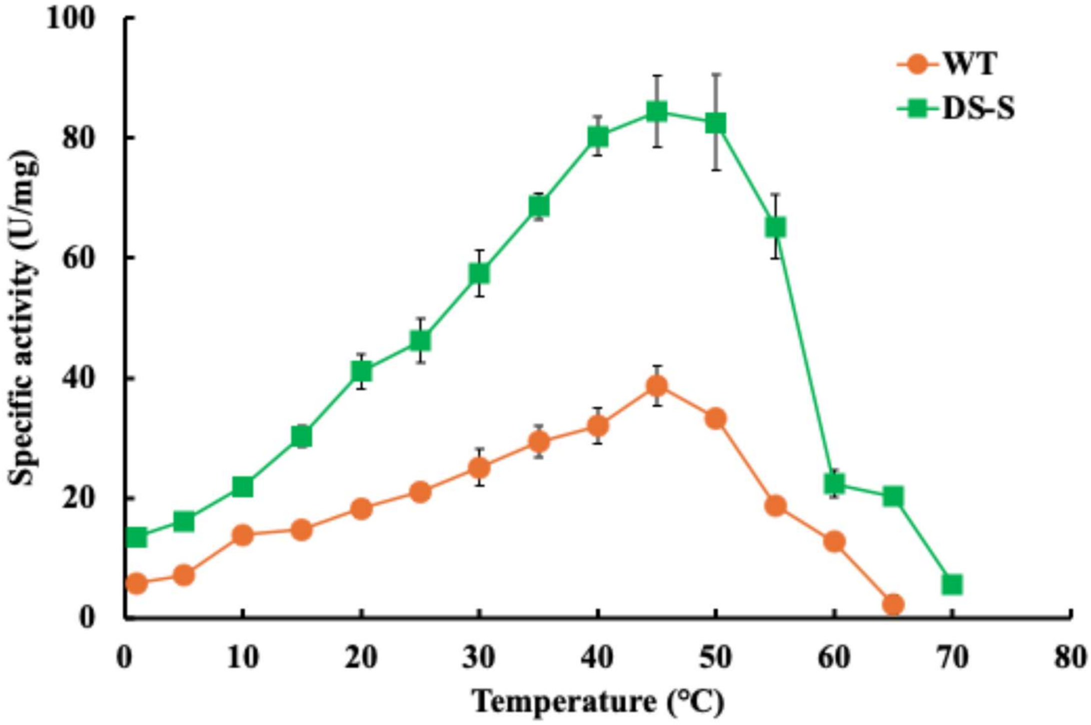

Supplement: Supplementary file 1 — Table S1. Thermal deactivation constants (k d) (min−1). Table S2. Kinetic constants of glucokinase from Pseudoalteromonas sp. AS‐131 (PsGK) C325S and glucokinase from Escherichia coli (EcGK) C20S C65S S309C (DS‐S). Fig. S1. Analytical anion exchange chromatography of (a) glucokinase from Pseudoalteromonas sp. AS‐131 (PsGK) wild‐type (WT) and (b) glucokinase from Escherichia coli (EcGK) WT using HiTrap Q HP. Fig. S2. Sodium dodecyl sulfate‐polyacrylamide gel electrophoresis (SDS/PAGE) analysis of glucokinase from Pseudoalteromonas sp. AS‐131 (PsGK) wild‐type (WT) and glucokinase from Escherichia coli (EcGK) WT. Fig. S3. Analytical gel filtration of glucokinase from Pseudoalteromonas sp. AS‐131 (PsGK) wild‐type (WT) and glucokinase from Escherichia coli (EcGK) WT. Fig. S4. Plot of relative activity of glucokinase from Pseudoalteromonas sp. AS‐131 (PsGK) wild‐type (WT) and glucokinase from Escherichia coli (EcGK) WT as a function of temperature. Fig. S5. Arrhenius plot of the substrate turnover (k cat) of glucokinase from Pseudoalteromonas sp. AS‐131 (PsGK) and glucokinase from Escherichia coli (EcGK). Fig. S6. Thermal stability of glucokinase from Pseudoalteromonas sp. AS‐131 (PsGK) wild‐type (WT) and glucokinase from Escherichia coli (EcGK) WT. Fig. S7. Residual activities of glucokinase from Pseudoalteromonas sp. AS‐131 (PsGK) wild‐type (WT) and glucokinase from Escherichia coli (EcGK) WT. Fig. S8. Disulfide bond and hydrogen bonds. Fig. S9. Plot of specific activity and relative activity of glucokinase from Pseudoalteromonas sp. AS‐131 (PsGK) wild‐type (WT) and PsGK C325S as a function of temperature. Fig. S10. The distance between cysteine sulfur (SG) atoms. Fig. S11. Circular dichroism (CD) spectrum of glucokinase from Escherichia coli (EcGK) wild‐type (WT), EcGK C20S C65S H312C (DS‐H), EcGK C20S C65S L313C (DS‐L), and EcGK C20S C65S S309C (DS‐S) at 20 °C. Fig. S12. Principal component analysis (PCA) projection of glucokinase from Escherichia coli (EcGK) wild‐typ [file FEBS-293-2617-s001.pdf]
